# H-Bond Mediated Phase-Transfer Catalysis: Enantioselective Generating of Quaternary Stereogenic Centers in β-Keto Esters

**DOI:** 10.3390/molecules27082508

**Published:** 2022-04-13

**Authors:** Patryk Niedbała, Maciej Majdecki, Piotr Grodek, Janusz Jurczak

**Affiliations:** Institute of Organic Chemistry PAS, 01-224 Warsaw, Poland; pniedbala@icho.edu.pl (P.N.); mmajdecki@icho.edu.pl (M.M.); pgrodek@icho.edu.pl (P.G.)

**Keywords:** phase-transfer catalysis, organocatalysis, enantioselectivity, *Cinchona* catalysts, alkylation

## Abstract

In this work, we would like to present the development of a highly optimized method for generating the quaternary stereogenic centers in β-keto esters. This enantioselective phase-transfer alkylation catalyzed by hybrid *Cinchona* catalysts allows for the efficient generation of the optically active products with excellent enantioselectivity, using only 1 mol% of the catalyst. The vast majority of phase-transfer catalysts in asymmetric synthesis work by creating ionic pairs with the nucleophile-attacking anionic substrate. Therefore, it is a sensible approach to search for new methodologies capable of introducing functional groups into the precursor’s structure, maintaining high yields and enantiomeric purity.

## 1. Introduction

The generation of a quaternary stereogenic center in organic molecules has for many years been a demanding challenge that fits into the theme of organic catalysis, even in the asymmetric variant [1,2,3,4,5,6]. The development of enantioselective synthetic methods, including organocatalysis, has led to the possibility of obtaining synthetic mimetics of compounds of natural origin [7,8,9,10,11]. The presence of a quaternary carbon center in a molecule is very often a key factor in the biological activity of natural products or drugs [12,13,14,15,16,17].

Among the possible substrates, β-dicarbonyl compounds, especially β-ketoesters, undoubtedly stand out [18,19,20]. These compounds can undergo numerous organic transformations (including alkylation) to form a quaternary stereogenic center with a variety of substituents, allowing further organic transformations and various post-functionalization processes [21,22,23]. Optically active α-alkylated β-dicarbonyl compounds are common building blocks of many natural compounds and pharmaceuticals.

Importantly, this type of derivatives can also provide an important direction for the synthesis of unnatural β-amino acids and other important building blocks [24,25,26]. Despite these important advantages, asymmetric synthesis using organocatalysts is still a major challenge for synthetic chemists. Therefore, the development of so-called “green” synthetic methods remains an important achievement [27,28]. To date, asymmetric α-alkylation reactions of β-ketoesters have been catalyzed mainly by palladium and enamine catalysts [29,30,31].

Among organocatalytic methods, phase transfer catalytic (PTC) reactions represent one of the simplest and most efficient tools for enantio-differentiation synthesis [32,33,34,35]. The most commonly used chiral catalysts of natural origin are ammonium salts, derivatives of Cinchona alkaloids, among others, due to their availability and economic considerations [36,37,38,39]. Moreover, it has been repeatedly shown that the use of large substituents on the nitrogen atom of the quinuclidinium ring improves the properties of such a catalyst [40,41].

To date, there are few examples in the literature of the use of PTC in the alkylation of β-ketoesters, which was initially limited mainly to phosphonium salts. One of the first reports dates back to the late 20th century [42]. The diamide catalysts synthesized by Manabe showed limited catalytic properties, with enantiomeric excesses as high as 50% *ee*. A few years later, Maruoka [43] and coworkers used quaternary ammonium catalysts built on a binaphthalene platform. These highly specialized catalysts showed high activity in the alkylation reactions of cyclic β-ketoesters as well as in the Michael reaction. The first examples of the use of Cinchona derivatives as catalysts in β-keto esters alkylation were presented by the Dehmlow [44] and Chinchilla [45] groups, but in both cases the maximum enantiomeric excess oscillated around 50% *ee*. To improve the enantiomeric excess, Kim [46] and coworkers synthesized catalysts containing phenyl rings substituted with tert-butyl groups. This type of substituent provides high steric hindrance. On the other hand, *N*-substituted *Cinchona* catalysts allowing high enantiomeric excesses >90% *ee* were reported in 2019 [47]. These catalysts have been used in alkylation reactions of β-ketoesters and β-ketoamides. Recently, our team developed and commercialized a family of novel hybrid quaternary ammonium salts based on *Cinchona* alkaloids [48,49,50] which are capable of catalyzing a range of organic reactions, for example, the quaternization of β-ketoesters by introducing a chlorine atom into the product structure [51]. The catalysts of this type are based on the structure of alkaloids of natural origin, which perfectly fits into the trends of the so-called “green chemistry”. Moreover, their advantage over the catalysts presented earlier in the literature consists in the trivial two-step synthesis, which allows to obtain pure catalysts in crystalline form at any scale with total excellent yields. After the successful application of our catalysts in chlorination, we decided to test other reactions and the choice fell on the alkylation of cyclic β-ketoesters, namely various indanone esters and cyclopentanones.

## 2. Results

**Figure 1:**
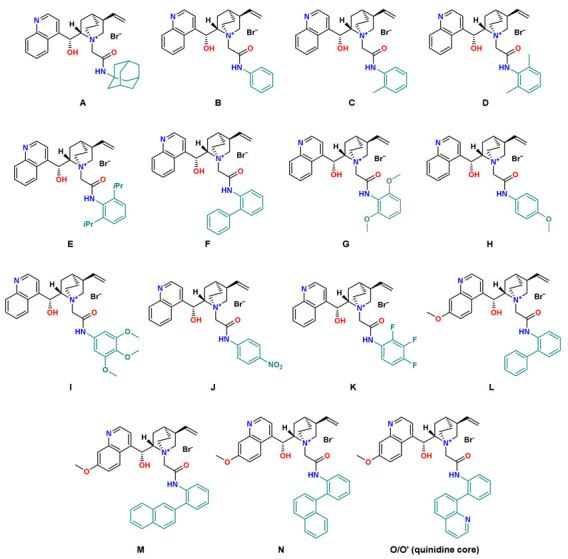
Cinchona phase-transfer catalysts **A**–**O**.

For several years, we have been presenting a new family of hybrid Cinchona catalysts which, in addition to the standard properties presented by phase-transfer catalysts, possess hydrogen bond donors in their structure which efficiently supports the generation of high enantiomeric excess (Figure 1) [48,49]. In addition, the use of aromatic substituents with appropriate geometry makes it possible to create other non-covalent interactions (e.g., π-π stacking) which favors the preorganization of the substrate [39]. Thus, we have presented a family of catalysts effective in the reactions of alkylation of glycine derivatives [48], epoxidation of α,β-unsaturated ketones [50], and recently also in α-chlorination of β-keto esters [51]. In this paper, we present the development of our methodology for another reaction, namely, the alkylation of β-keto esters with the formation of a quaternary stereogenic center.

First, using catalyst **L** which showed the highest efficiency in our previous studies, we optimized the β-keto ester **1a** alkylation procedure. We screened several bases in toluene/CHCl_3_ [7:3, *v*/*v*] mixture (Table 1, entries 1–6). In almost all cases, the product was received in quantitative yield. Using solid bases, as well as 50% aqueous solutions, provided similar results, however the best ones occurred for solid KF. Next, we screened the impact of the solvent (Table 1, entries 7–11). As in our previous papers, the best results occurred for the toluene/CHCl_3_ [7:3, *v*/*v*] mixture. It is worth mentioning that the reaction enantioselectivity was improved to 72% *ee* when the temperature was decreased to 5 °C. Moreover, the catalyst loading equal to only 1 mol% was enough to effectively carry out the reaction. Finally, the optimal conditions are the reaction carried out in the toluene/CHCl_3_ mixture [7:3, *v*/*v*], 1 mol% of catalyst, 5 °C, 2 eq of base (KF), and 1.2 eq of the alkylating agent.

Next, using the model β-keto ester **1a**, we studied the modification of the catalyst structure, in particular the substituent at the amide group. The only catalyst in our list with an aliphatic substituent **A** (adamantyl ring) allowed us to obtain product **2a** with an enantiomeric excess of 60% *ee* (Table 2, entry 1). The simplest of the catalysts, with phenyl substituent **B**, as well as the ortho-substituted catalysts **C**–**E** did not increase the enantiomeric excess (55–61% *ee*) (Table 2, entries 3–5). The situation was similar after the introduction of both electron-donating methoxy groups (catalysts **G**–**I**, 31–42% *ee*, Table 1, entries 8–10), as well as in the presence of electron-withdrawing groups (catalyst **J** with –NO_2_ group, 56% *ee* and 2,3,4-trifluorophenyl **K**, 57% *ee*). Slightly better enantiomeric excesses were obtained for compounds with biphenyl substituent—**F** (64% *ee*, Table 1, entry 6). In all cases, we observed complete conversion of the substrate within 3–7 h.

Then, we decided to check the activity of catalysts constructed with a quinine core. This procedure led to a series of reactions in which we were able to obtain higher enantiomeric excesses. The catalyst with the biphenyl substituent adjacent to the amide group **L** allowed us to obtain the product with 68% *ee* (Table 2, entry 13). Compounds substituted in ortho-position of the aromatic ring with α- and β-naphthyl rings **M** and **N** gave 73% and 70% *ee*, respectively. The highest catalytic activity was obtained for the catalyst substituted with quinoline in ortho-position **O** (80% *ee*, Table 2, entry 16). The use of catalysts with a quinine platform allowed us to shorten the reaction time to 3–4 h. At the same time, it is worth noting that in all presented cases the reactions occurred with almost quantitative yields (96–99%).

After the experiments that led to the determination of the best catalyst, we decided to check the efficiency of the reactions by increasing the steric hindrance of the ester group (isopropyl, tert-butyl) and changing the geometric structure of the substrate (indanone, cyclopentanone), using catalyst **O**. In reactions with indanone derivatives **1a**–**c**, we observed that increasing the steric hindrance favors the improvement of the enantiomeric excess, with the highest values for the tert-butyl **1c** (91% *ee*) ester (Table 3, entry 3). The same occurred for cyclopentanone derivatives **1d** and **1e**: increased steric hindrance resulted in higher asymmetric induction (Table 3, entries 4 and 5). We also performed an additional comparative experiment for the methyl ester using a quinidine catalyst. This procedure allowed for an almost complete reversal of the enantiomeric excess (80 vs. –84% *ee*).

In the next stage, we decided to investigate the influence of the nature of the electrophile used in the reaction. In this series of reactions, we used indanone tert-butyl ester **1c** as the optimal β-keto ester substrate. The model benzyl bromide as well as isomers of methyl benzyl bromide allowed us to obtain products **2c** and **3**–**6** with 89–91% *ee* (Table 4, entries 1–4). The presence of chloride in the electrophile molecule **6** did not significantly change the enantiomeric excess (88% *ee*, Table 4, entry 5).

**Figure 2:**
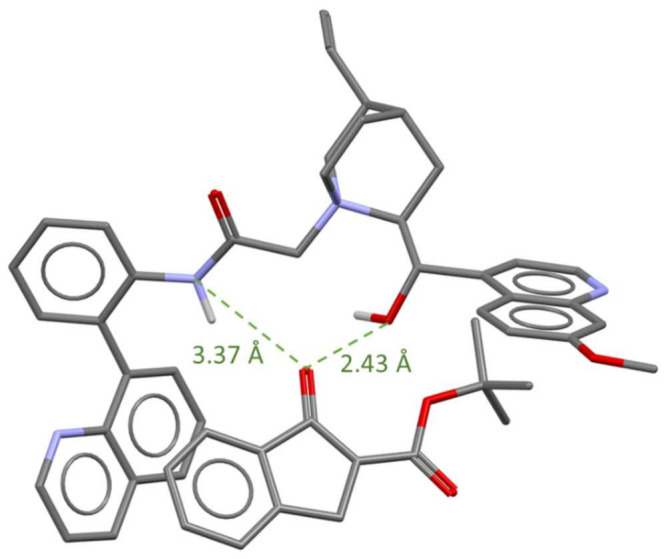
Model of a possible intermediate state for the reaction.

To gain a better insight into the complexing process, we decided to conduct computational studies indicating a plausible intermediate state (Figure 2). After the first step, namely the deprotonation of the substrate, the complex of phase-transfer catalyst and enolate is formed. The nucleophilic substrate can be stabilized by two intermolecular hydrogen bonds: with amide function of the catalyst and in addition with the hydroxyl group. A key element determining the high enantioselectivity is the quinoline ring which blocks the re-face of the enolate. These interactions synergistically stabilize the complex and further increase the selectivity of the underlying nucleophile attack. Conducted computational studies are in agreement with the obtained results. The lowest energy conformation of the complex of catalyst with enolate was found after conducting a conformational search analysis and selected conformers with the lowest energies which were then optimized without any constrains at DFT/M06-2X/6-31G(d) level of theory using program Spartan’18 Parallel Suite [52,53,54,55,56].

## 3. Materials and Methods

### 3.1. Reagents and General Methods

All reagents were used as received. The solvents were dried by distillation over the appropriate drying agents. All solvents were obtained from common suppliers and used as received. TLC was carried out on Merck Kieselgel F254 plates. Melting points were determined using a Boëtius M HMK hot-stage apparatus and were uncorrected. The NMR spectra were recorded on a Bruker Mercury 400 MHz and Bruker 500 MHz and Varian 600 MHz instruments (see SI). Chemical shifts are reported in ppm (δ) and are set to the solvent residue peak. J coupling constants values are re-ported in Hz. Mass spectral analyses were performed with the ESI-TOF technique on a Mariner mass spectrometer from PerSeptive Biosystem. The enantiomeric excesses of products were determined by chiral HPLC analysis using Chiralcel AD-H column (see Supplementary Materials).

Amide-based Cinchona catalysts **A**–**L** were prepared according to our previous procedure [48]. Catalysts **M**-**O** have not been previously reported. Alkylated indanone and cyclopentanone derivatives **2a**–**e** and **4** are known from the literature and their analytical data fully matched those reported previously in the literature [43,45,47].

General procedure for the asymmetric alkylation of β-keto esters **1a–e**. A mixture of the appropriate β-keto ester **1a**–**e** (0.2 mmol), catalyst (0.002 mmol), and KF (0.4 mmol) was stirred in toluene/CHCl_3_ [7:3, *v*/*v*] for 30 min. Subsequently, the mixture was cooled to 5 °C and alkylating agent (0.24 mmol) was added in one portion. The reaction was mixed for 4 h. Then, the mixture was filtered through a short pad of silica and eluted using hexane/ethyl acetate [8:2, *v*/*v*]. The organic solvents were evaporated under reduced pressure to obtain a pure product **2a**–**e** and **3**–**6** in the reported yields and enantiopurities.

### 3.2. Synthetic Procedures

#### 3.2.1. Synthesis of (1S,2S,4S,5R)-5-Ethenyl-2-[(R)-hydroxy(7-methoxyquinolin-4-yl)methyl]-1-({[2-(naphthalen-2-yl)phenyl]carbamoyl}methyl)-1-azabicyclo [2.2.2]octan-1-ium bromide (**M**)

Following the literature procedure [48] and using the corresponding bromoamide (1.0 g, 2.9 mmol), the catalyst **M** (1.9 g, 2.9 mmol, 97%) was obtained as colorless powder (m.p. 122–123 °C). ^1^H NMR (400 MHz, DMSO-*d*_6_): δ 10.21 (s, 1H), 8.77 (d, *J* = 4.2 Hz, 1H), 7.98–7.90 (m, 3H), 7.68 (d, *J* = 6.6 Hz, 2H), 7.60–7.28 (m, 10H), 6.63 (s, 1H), 6.56 (s, 1H), 5.91 (s, 1H), 5.82 (s, 1H), 5.71–5.42 (m, 1H), 5.12 (d, *J* = 13.9 Hz, 1H), 5.00 (d, *J* = 14,1 Hz) 4.82 (t, *J* = 14.9 Hz, 1H), 4.43–4.21 (m, 1H), 4.37–4.22 (m, 2H), 4.06 (dd, *J* = 15.9, 7.6 Hz, 1H), 3.74 (s, 3H), 3.12 (t, *J* = 11.3 Hz, 2H), 2.68 (s, 1H), 2.59 (s, 1H), 1.98 (s, 2H), 1.88–1.76 (m, 2H). ^13^C{^1^H} NMR (101 MHz, DMSO-*d*_6_): δ 163.2, 157.9, 150.3, 147.2, 145.4, 143.7, 143.3, 138.0, 136.4, 134.3, 131.4, 130.7, 128.4, 128.2, 127.7, 126.2, 125.9, 125.4, 122.2, 121.5, 120.2, 115.5, 100.9, 65.7, 63.0, 59.6, 58.7, 56.5, 56.0, 36.6, 25.3, 24.7, 21.2. HRMS ESI (*m*/*z*): calc for C_38_H_38_N_3_O_3_ [M]^+^: 584.2913, found: 584.2919.

#### 3.2.2. Synthesis of (1S,2S,4S,5R)-5-Ethenyl-2-[(R)-hydroxy(7-methoxyquinolin-4-yl)methyl]-1-({[2-(naphthalen-1-yl)phenyl]carbamoyl}methyl)-1-azabicyclo [2.2.2]octan-1-ium bromide (**N**)

Following the literature procedure [48] and using the corresponding bromoamide (1.0 g, 2.9 mmol), the catalyst **N** (1.8 g, 2.8 mmol, 95%) was obtained as colorless powder (m.p. 135–136 °C). ^1^H NMR (500 MHz, DMSO-*d*_6_): δ 10.22 (d, *J* = 6.7 Hz, 1H), 8.79–8.75 (m, 1H), 7.98–7.92 (m, 3H), 7.72–7.66 (m, 2H), 7.61–7.27 (m, 10H), 6.61 (d, *J* = 3.5 Hz, 1H), 5.91 (s, 1H), 5.82 (s, 1H), 5.70–5.43 (m, 1H), 5.10 (d, *J* = 13.9 Hz, 1H), 4.98 (d, *J* = 12.6 Hz, 1H), 4.82 (dd, *J* = 20.7, 13.8 Hz, 1H), 4.44–4.17 (m, 1H), 4.37–4.21 (m, 2H), 4.06 (dd, *J* = 16.1, 10.8 Hz, 1H), 3.74 (s, 3H), 3.32–3.07 (m, 2H), 2.66 (s, 1H), 2.58 (s, 1H), 2.05–1.94 (m, 2H), 1.84–1.77 (m, 2H). ^13^C{^1^H} NMR (126 MHz, DMSO-*d*_6_): δ 163.6, 163.4, 157.9, 147.3, 143.7, 143.4, 137.7, 135.8, 134.3, 133.4, 131.4, 131.2, 128.3, 127.9, 127.5, 127.1, 127.0, 126.8, 126.2, 125.8, 125.5, 125.2, 125.0, 122.1, 120.3, 115.4, 101.0, 65.6, 63.6, 63.1, 59.3, 58.7, 56.3, 56.0, 36.5, 25.2, 24.7, 21.2. HRMS ESI (*m*/*z*): calc for C_38_H_38_N_3_O_3_ [M]^+^: 584.2913, found: 584.2915.

#### 3.2.3. (1S,2S,4S,5R)-5-Ethenyl-2-[(R)-hydroxy(7-methoxyquinolin-4-yl)methyl]-1-({[2-(quinolin-8-yl)phenyl]carbamoyl}methyl)-1-azabicyclo [2.2.2]octan-1-ium bromide (**O**)

Following the literature procedure [48] and using the corresponding bromoamide (1.0 g, 2.9 mmol), the catalyst **O** (1.9 g, 2.9 mmol, 98%) was obtained as colorless powder (m.p. 150–151 °C). ^1^H NMR (600 MHz, DMSO-*d*_6_): δ 10.01 (s, 1H), 8.79 (d, *J* = 4.5 Hz, 2H), 8.41 (d, *J* = 6.3 Hz, 1H), 8.02 (d, *J* = 8.1 Hz, 1H), 7.96 (d, *J* = 9.2 Hz, 1H), 7.76–7.63 (m, 4H), 7.53–7.47 (m, 3H), 7.45–7.37 (m, 3H), 6.65 (s, 1H), 5.96–5.88 (m, 2H), 5.21 (dd, *J* = 17.6, 14.0 Hz, 2H), 4.41 (d, *J* = 15.7 Hz, 1H), 4.30 (t, *J* = 9.1 Hz, 1H), 4.26 (t, *J* = 9.2 Hz, 1H), 4.04–3.95 (m, 1H), 3.73 (s, 4H), 3.44 (s, 1H), 3.13 (d, *J* = 9.6 Hz, 1H), 2.70 (d, *J* = 7.2 Hz, 1H), 2.01 (d, *J* = 10.2 Hz, 1H), 1.89–1.75 (m, 3H), 0.89–0.83 (m, 1H). ^13^C{^1^H} NMR (151 MHz, DMSO-*d*_6_): δ 163.1, 157.9, 150.3, 147.2, 145.5, 143.7, 143.3, 136.5, 136.4, 134.4, 131.3, 130.9, 128.4, 128.2, 127.6, 126.3, 125.7, 122.1, 121.5, 120.4, 117.1, 101.6, 66.0, 63.8, 60.1, 59.0, 56.5, 56.0, 37.1, 26.2, 22.8, 20.4. HRMS ESI (*m*/*z*): calc for C_37_H_37_N_4_O_3_ [M]^+^: 585.2866, found: 585.2859.

#### 3.2.4. Methyl 2-Benzyl-1-oxo-2,3-dihydro-1H-indene-2-carboxylate (**2a**)

Following the general procedure, the product **2a** (55 mg, 0.2 mmol, 99%, 80% *ee*) was obtained as colorless oil. ^1^H NMR (500 MHz, CDCl_3_) δ 7.72 (d, *J* = 7.7 Hz, 1H), 7.51 (t, *J* = 7.4 Hz, 1H), 7.35–7.29 (m, 2H), 7.19–7.09 (m, 5H), 3.70 (s, 3H), 3.61 (d, *J* = 17.3 Hz, 1H), 3.47 (d, *J* = 14.0 Hz, 1H), 3.28 (d, *J* = 14.0 Hz, 1H), 3.15 (d, *J* = 17.3 Hz, 1H). ^13^C{^1^H} NMR (126 MHz, CDCl_3_) δ 202.2, 171.3, 153.3, 136.5, 135.4, 135.3, 130.1, 128.4, 127.8, 127.0, 126.4, 124.8, 61.8, 53.0, 39.9, 35.5. HPLC-separation conditions: Chiralcel AD-H, 20 °C, 254 nm, hexane/*i*PrOH [98:2, *v*/*v*], 0.8 mL/min; t_major_ = 11.7 min, t_minor_ = 9.0 min. Analytical data fully matched those reported previously in the literature [45,47].

#### 3.2.5. Propan-2-yl 2-Benzyl-1-oxo-2,3-dihydro-1H-indene-2-carboxylate (**2b**)

Following the general procedure, the product **2b** (60 mg, 0.2 mmol, 98%, 85% *ee*) was obtained as colorless oil. ^1^H NMR (400 MHz, CDCl_3_) δ 7.73 (d, *J* = 7.7 Hz, 1H), 7.52 (t, *J* = 7.4 Hz, 1H), 7.32 (dd, *J* = 12.8, 7.4 Hz, 2H), 7.19–7.10 (m, 5H), 5.09–4.96 (m, 1H), 3.61 (d, *J* = 17.3 Hz, 1H), 3.48 (d, *J* = 14.1 Hz, 1H), 3.27 (d, *J* = 14.1 Hz, 1H), 3.15 (d, *J* = 17.3 Hz, 1H), 1.18 (dd, *J* = 6.3, 2.4 Hz, 6H). ^13^C{^1^H} NMR (101 MHz, CDCl_3_) 202.4, 170.3, 153.4, 136.7, 135.4, 135.3, 130.1, 128.3, 127.6, 126.8, 126.3, 124.7, 69.5, 61.9, 39.7, 35.6, 21.7, 21.6. HPLC-separation conditions: Chiralcel AD-H, 20 °C, 254 nm, hexane/*i*PrOH [98:2, *v*/*v*], 0.8 mL/min; t_major_ = 14.4 min, t_minor_ = 15.8 min. Analytical data fully matched those reported previously in the literature [47].

#### 3.2.6. Tert-Butyl 2-Benzyl-1-oxo-2,3-dihydro-1H-indene-2-carboxylate (**2c**)

Following the general procedure, the product **2c** (64 mg, 0.2 mmol, 99%, 91% *ee*) was obtained as colorless oil. For the reaction using 1 mmol of **1c** (232 mg), the product **2c** was obtained in an amount of 319 mg (1.0 mmol, 99%). ^1^H NMR (500 MHz, CDCl_3_) δ 7.72 (d, *J* = 7.6 Hz, 1H), 7.51 (t, *J* = 7.4 Hz, 1H), 7.32 (dd, *J* = 14.0, 7.3 Hz, 2H), 7.20–7.10 (m, 5H), 3.57 (d, *J* = 17.1 Hz, 1H), 3.44 (d, *J* = 14.1 Hz, 1H), 3.27 (d, *J* = 14.1 Hz, 1H), 3.12 (d, *J* = 17.1 Hz, 1H), 1.38 (s, 9H). ^13^C{^1^H} NMR (126 MHz, CDCl_3_) δ 202.8, 169.9, 153.5, 137.0, 135.6, 135.1, 130.1, 128.3, 127.6, 126.8, 126.2, 124.6, 82.2, 62.6, 39.5, 35.8, 27.9. HPLC-separation conditions: Chiralcel AD-H, 20 °C, 254 nm, hexane/*i*PrOH [98:2, *v*/*v*], 0.8 mL/min; t_major_ = 6.1 min, t_minor_ = 5.5 min. Analytical data fully matched those reported previously in the literature [47].

#### 3.2.7. Methyl 1-Benzyl-2-oxocyclopentane-1-carboxylate (**2d**)

Following the general procedure, the product **2d** (46 mg, 0.2 mmol, 99%, 61% *ee*) was obtained as colorless oil. ^1^H NMR (600 MHz, CDCl_3_) δ 7.21–7.17 (m, 2H), 7.17–7.13 (m, 1H), 7.05 (d, *J* = 7.0 Hz, 2H), 3.65 (s, 3H), 3.14 (d, *J* = 13.8 Hz, 1H), 3.04 (d, *J* = 13.8 Hz, 1H), 2.38–2.27 (m, 2H), 2.01–1.94 (m, 1H), 1.92–1.86 (m, 1H), 1.85–1.78 (m, 1H), 1.57–1.49 (m, 1H). ^13^C{^1^H} NMR (151 MHz, CDCl_3_) δ 215.0, 171.5, 136.6, 130.3, 128.5, 127.0, 61.6, 52.8, 39.3, 38.5, 31.8, 19.6. HPLC-separation conditions: Chiralcel AD-H, 20 °C, 254 nm, hexane/*i*PrOH [98:2, *v*/*v*], 0.8 mL/min; t_major_ = 7.1 min, t_minor_ = 8.1 min. Analytical data fully matched those reported previously in the literature [47].

#### 3.2.8. Tert-Butyl 1-Benzyl-2-oxocyclopentane-1-carboxylate (**2e**)

Following the general procedure, the product **2e** (53 mg, 0.2 mmol, 96%, 74% *ee*) was obtained as colorless oil. ^1^H NMR (500 MHz, CDCl_3_) δ 7.20–7.15 (m, 2H), 7.15–7.11 (m, 1H), 7.07 (d, *J* = 6.9 Hz, 2H), 3.05 (s, 2H), 2.32–2.24 (m, 2H), 1.92–1.77 (m, 3H), 1.53–1.45 (m, 1H), 1.36 (s, 9H). ^13^C{^1^H} NMR (126 MHz, CDCl_3_): δ 215.5, 170.5, 137.1, 130.5, 128.4, 126.8, 82.1, 62.1, 38.9, 38.4, 32.1, 28.0, 19.6. HPLC-separation conditions: Chiralcel AD-H, 20 °C, 254 nm, hexane/*i*PrOH [98:2, *v*/*v*], 0.8 mL/min; t_major_ = 8.2 min, t_minor_ = 9.6 min. Analytical data fully matched those reported previously in the literature [43].

#### 3.2.9. Tert-butyl(2R)-2-[(2-methylphenyl)methyl]-1-oxo-2,3-dihydro-1H-indene-2-carboxylate (**3**)

Following the general procedure, the product **3** (66 mg, 0.2 mmol, 98%, 90% *ee*) was obtained as colorless oil. ^1^H NMR (500 MHz, CDCl_3_) δ 7.77 (d, *J* = 7.6 Hz, 1H), 7.54 (t, *J* = 7.1 Hz, 1H), 7.35 (t, *J* = 7.9 Hz, 2H), 7.12–6.98 (m, 4H), 3.71 (d, *J* = 17.1 Hz, 1H), 3.58 (d, *J* = 15.3 Hz, 1H), 3.19 (d, *J* = 15.3 Hz, 1H), 2.99 (d, *J* = 17.0 Hz, 1H), 2.28 (s, 3H), 1.36 (s, 9H). ^13^C{^1^H} NMR (126 MHz, CDCl_3_) δ 203.0, 170.0, 153.7, 137.1, 135.9, 135.2, 130.4, 128.9, 127.6, 126.6, 126.3, 126.0, 124.7, 82.2, 62.3, 36.1, 35.6, 27.8, 20.3. HPLC-separation conditions: Chiralcel AD-H, 20 °C, 254 nm, hexane/*i*PrOH [98:2, *v*/*v*], 0.8 mL/min; t_major_ = 9.6 min, t_minor_ = 8.6 min. HRMS ESI (m/z): calc for C_22_H_24_O_3_Na [M + Na]^+^: 359.1623, found: 359.1615.

#### 3.2.10. Tert-Butyl 2-[(3-Methylphenyl)methyl]-1-oxo-2,3-dihydro-1H-indene-2-carboxylate (**4**)

Following the general procedure, the product **4** (65 mg, 0.2 mmol, 97%, 91% *ee*) was obtained as colorless oil. ^1^H NMR (400 MHz, CDCl_3_) δ 7.73 (d, *J* = 7.7 Hz, 1H), 7.54–7.49 (m, 1H), 7.33 (dd, *J* = 14.5, 7.4 Hz, 2H), 7.06 (t, *J* = 7.5 Hz, 1H), 6.99–6.92 (m, 3H), 3.57 (d, *J* = 17.2 Hz, 1H), 3.43 (d, *J* = 14.1 Hz, 1H), 3.19 (d, *J* = 14.1 Hz, 1H), 3.11 (d, *J* = 17.2 Hz, 1H), 2.24 (s, 3H), 1.39 (s, 9H). ^13^C{^1^H} NMR (101 MHz, CDCl_3_) δ 202.7, 169.8, 153.5, 137.8, 137.0, 135.6, 135.1, 130.9, 128.2, 127.5, 127.5, 127.1, 126.3, 124.6, 82.2, 62.7, 39.5, 35.8, 27.9, 21.5. HPLC-separation conditions: Chiralcel AD-H, 20 °C, 254 nm, hexane/*i*PrOH [98:2, *v*/*v*], 0.8 mL/min; t_major_ = 7.9 min, t_minor_ = 7.2 min. HRMS ESI (m/z): calc for C_22_H_24_O_3_Na [M + Na]^+^: 359.1623, found: 359.1613. Analytical data fully matched those reported previously in the literature [45].

#### 3.2.11. Tert-Butyl 2-[(4-Methylphenyl)methyl]-1-oxo-2,3-dihydro-1H-indene-2-carboxylate (**5**)

Following the general procedure, the product **5** (65 mg, 0.2 mmol, 98%, 89% *ee*) was obtained as colorless oil. ^1^H NMR (500 MHz, CDCl_3_) δ 7.73 (d, *J* = 7.6 Hz, 1H), 7.51 (t, *J* = 7.3 Hz, 1H), 7.32 (dd, *J* = 15.2, 7.5 Hz, 2H), 7.04 (d, *J* = 7.9 Hz, 2H), 6.98 (d, *J* = 7.9 Hz, 2H), 3.54 (d, *J* = 17.2 Hz, 1H), 3.40 (d, *J* = 14.2 Hz, 1H), 3.22 (d, *J* = 14.2 Hz, 1H), 3.11 (d, *J* = 17.2 Hz, 1H), 2.24 (s, 3H), 1.38 (s, 9H). ^13^C{^1^H} NMR (126 MHz, CDCl_3_) δ 202.8, 169.9, 153.6, 136.3, 135.6, 135.1, 133.9, 130.0, 129.0, 127.5, 126.3, 124.6, 82.1, 62.7, 39.1, 35.7, 27.9, 21.1. HPLC-separation conditions: Chiralcel AD-H, 20 °C, 254 nm, hexane/*i*PrOH [98:2, *v*/*v*], 0.8 mL/min; t_major_ = 10.8 min, t_minor_ = 13.2 min. HRMS ESI (m/z): calc for C_22_H_24_O_3_Na [M + Na]^+^: 359.1623, found: 359.1621.

#### 3.2.12. Tert-Butyl 2-[(4-Chlorophenyl)methyl]-1-oxo-2,3-dihydro-1H-indene-2-carboxylate (**6**)

Following the general procedure, the product **6** (71 mg, 0.2 mmol, 97%, 88% *ee*) was obtained as colorless oil. ^1^H NMR (500 MHz, CDCl_3_) δ 7.72 (d, *J* = 7.7 Hz, 1H), 7.53 (t, *J* = 7.4 Hz, 1H), 7.36–7.31 (m, 2H), 7.14 (d, *J* = 8.4 Hz, 2H), 7.09 (d, *J* = 8.4 Hz, 2H), 3.54 (d, *J* = 17.2 Hz, 1H), 3.37 (d, *J* = 14.1 Hz, 1H), 3.25 (d, *J* = 14.2 Hz, 1H), 3.06 (d, *J* = 17.1 Hz, 1H), 1.37 (s, 9H). ^13^C{^1^H} NMR (126 MHz, CDCl_3_) δ 202.6, 169.8, 153.3, 135.5, 135.4, 135.3, 132.7, 131.5, 128.5, 127.7, 126.3, 124.7, 82.4, 62.3, 38.7, 35.8, 27.9. HPLC-separation conditions: Chiralcel AD-H, 20 °C, 254 nm, hexane/*i*PrOH [98:2, *v*/*v*], 0.8 mL/min; t_major_ = 14.5 min, t_minor_ = 11.0 min. HRMS ESI (m/z): calc for C_21_H_21_ClO_3_Na [M + Na]^+^: 379.1077, found: 379.1085.

## Figures and Tables

**Table 1:** Optimization of the reaction conditions for the alkylation of β-keto ester **1a** with phase-transfer catalyst **L** ^a^.

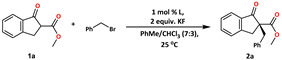					
Entry	Solvent	Base	T (°C)	Yield ^b^ (%)	*ee*^c^ (%)
1	PhMe/CHCl_3_ (7/3)	K_2_CO_3_	25	99	64
2	PhMe/CHCl_3_ (7/3)	50%_aq_ K_2_CO_3_	25	99	62
3	PhMe/CHCl_3_ (7/3)	KF	25	99	67
4	PhMe/CHCl_3_ (7/3)	50%_aq_ KF	25	99	64
5	PhMe/CHCl_3_ (7/3)	Na_2_CO_3_	25	95	65
6	PhMe/CHCl_3_ (7/3)	50%_aq_ Na_2_CO_3_	25	94	61
7	PhMe	KF	25	99	67
8	m-Xylene	KF	25	99	66
9	CH_2_Cl_2_	KF	25	99	66
10	CHCl_3_	KF	25	99	65
11	PhMe/CHCl_3_ (7/3)	KF	10	99	71
12	PhMe/CHCl_3_ (7/3)	KF	5	99	72

^a^ Unless otherwise specified, the reactions were performed with **1a** (1 equiv.), BnBr (1.25 equiv.), phase-transfer catalyst **L** (1 mol%), and base (2 equiv.). ^b^ Yields shown are of isolated products. ^c^ Determined by chiral HPLC (Chiralcel AD-H column).

**Table 2:** Screening of phase-transfer catalysts **A–O** using substrate **1a** ^a^.

Entry	Catalyst		Time (h)	Yield ^b^ (%)		*ee*^c^ (%)	
1	A		5	99		60	
2	B		6	98		55	
3	C		5	99		52	
4	D		5	99		60	
5	E		5	99		61	
6	F		4	99		64	
7	G		5	98		31	
8	H		6	96		49	
9	I		7	98		42	
10	J		7	97		56	
11	K		6	98		57	
12	L		4	99		68	
13	M		4	99		70	
14	N		4	99		73	
15	O	O’	3	99	99	80	−84

^a^ Unless otherwise specified, the reactions were performed with **1a** (1 equiv.), BnBr (1.25 equiv.), phase-transfer catalyst (1 mol%), and base (2 equiv.). ^b^ Yields shown are of isolated products. ^c^ Determined by chiral HPLC (Chiralcel AD-H column).

**Table 3:** Screening of β-keto esters **1a**–**e** ^a^.

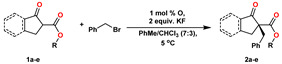			
Entry	Substrate	Yield ^b^ [%]	*ee*^c^ [%]
1	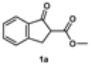	99	80
2	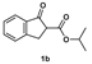	98	85
3	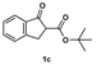	99	91
4	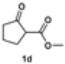	99	61
5	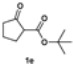	96	74

^a^ Unless otherwise specified, the reactions were performed with appropriate β-keto ester (1 equiv.), BnBr (1.25 equiv.), phase-transfer catalyst **O** (1 mol%), and base (2 equiv.). ^b^ Yields shown are of isolated products. ^c^ Determined by chiral HPLC (Chiralcel AD-H column).

**Table 4:** Screening of alkylating agents ^a^.

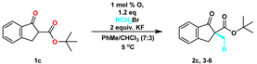			
Entry	Substrate	Yield ^b^ (%)	*ee* ^c^ (%)
1	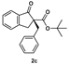	99	91
2	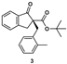	98	90
3	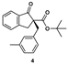	97	91
4	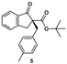	98	89
5	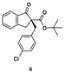	98	88

^a^ Unless otherwise specified, the reactions were performed with **1c** (1 equiv.), appropriate alkylating agent (1.25 equiv.), phase-transfer catalyst **O** (1 mol%), and base (2 equiv.). ^b^ Yields shown are of isolated products. ^c^ Determined by chiral HPLC (Chiralcel AD-H column).

## Data Availability

The data presented in this study are available on request from the corresponding author.

## Supplementary Materials

The following supporting information can be downloaded at: https://www.mdpi.com/article/10.3390/molecules27082508/s1: Copies of ^1^H and ^13^C NMR spectra of compounds synthesized, chromatograms of enantioenriched mixtures.
