# Supplementary material for: H-Bond Mediated Phase-Transfer Catalysis: Enantioselective Generating of Quaternary Stereogenic Centers in β-Keto Esters
Source: Molecules. 2022 Apr 13;27(8):2508. doi: 10.3390/molecules27082508 (PMC9024675; doi:10.3390/molecules27082508)
Supplement: Supplementary file 1 [file molecules-27-02508-s001.zip › molecules-1647424-supplementary.pdf]

H-bond Mediated Phase-Transfer Catalysis: Enantioselective Generating of Quaternary Stereogenic Centers in β-Keto Esters

Patryk Niedbała, Maciej Majdecki, Piotr Grodek, Janusz Jurczak*

Institute of Organic Chemistry, Polish Academy of Sciences, Kasprzaka 44/52, 01-224 Warsaw, Poland

E-mail: jurczak_group@icho.edu.pl

Contents

1. Copies of ^{1}H and ^{13}C NMR spectra 2
2. DFT studies and corresponding Cartesian coordinates 15
3. Copies of HPLC chromatograms 16

1. Copies of ^1H and ^{13}C NMR spectra

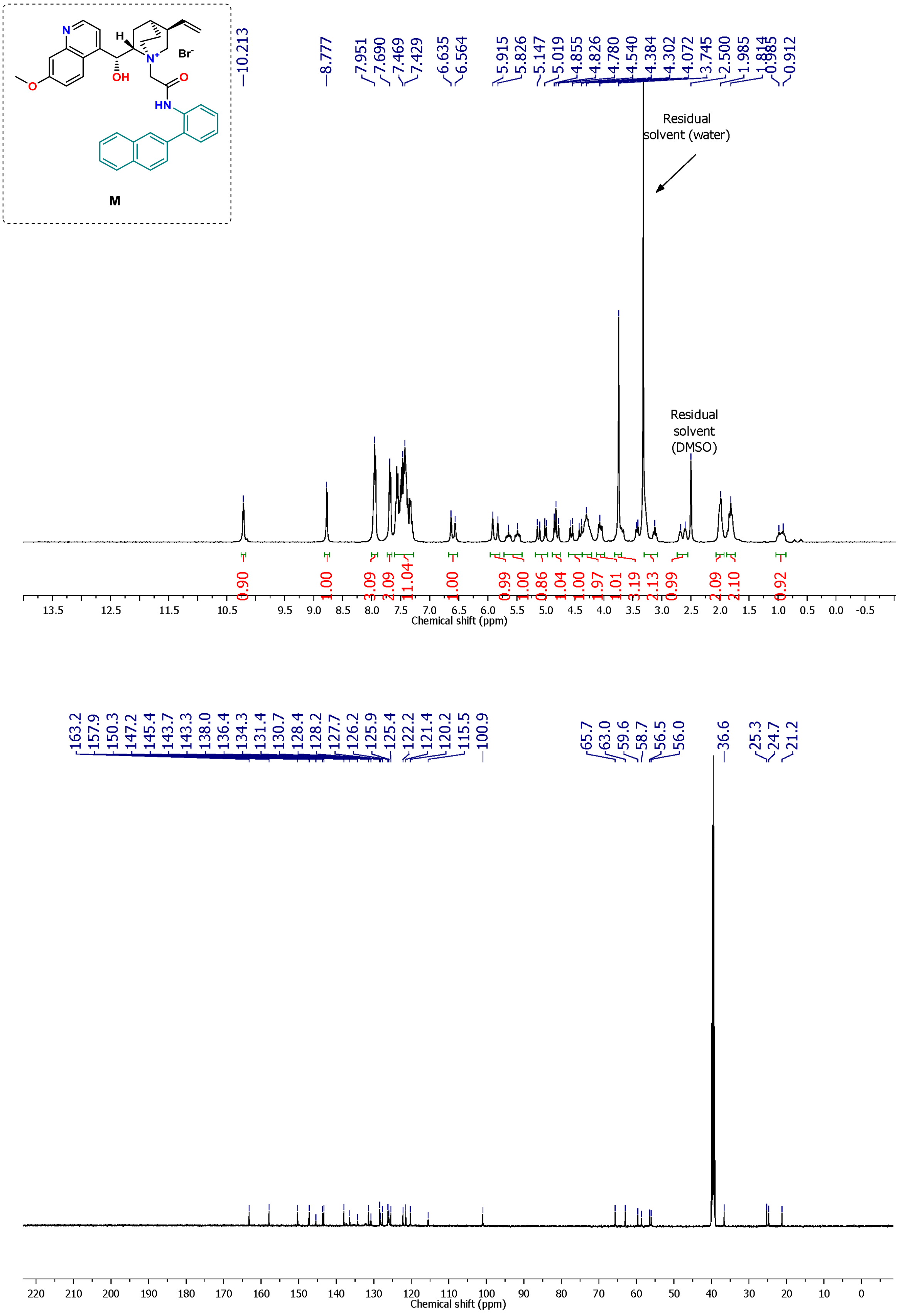

Figure S1. ^1H NMR (400 MHz) and ^{13}C NMR (101 MHz) spectra of compound **M** in DMSO-d_6.

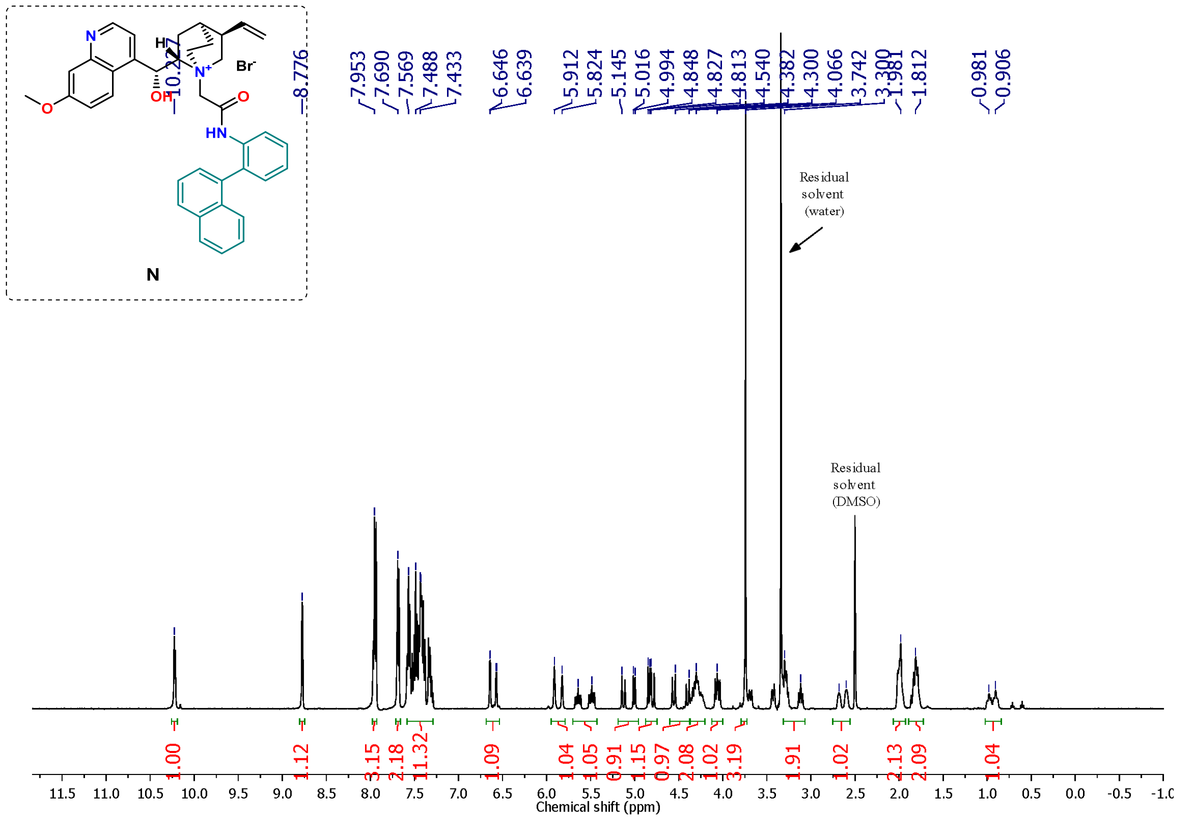

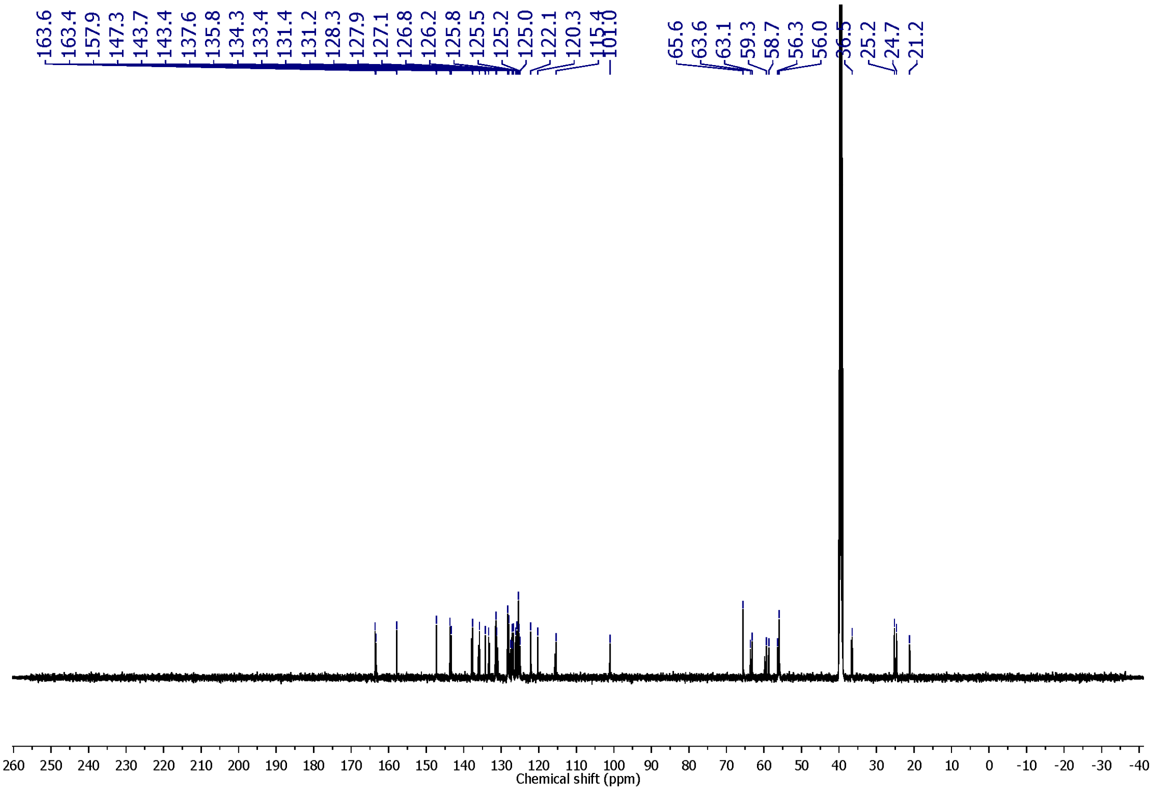

Figure S2. ^{1}H NMR (500 MHz) and ^{13}C NMR (126 MHz) spectra of compound **N** in DMSO-d_6.

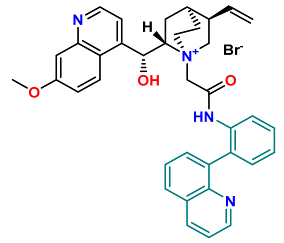

O

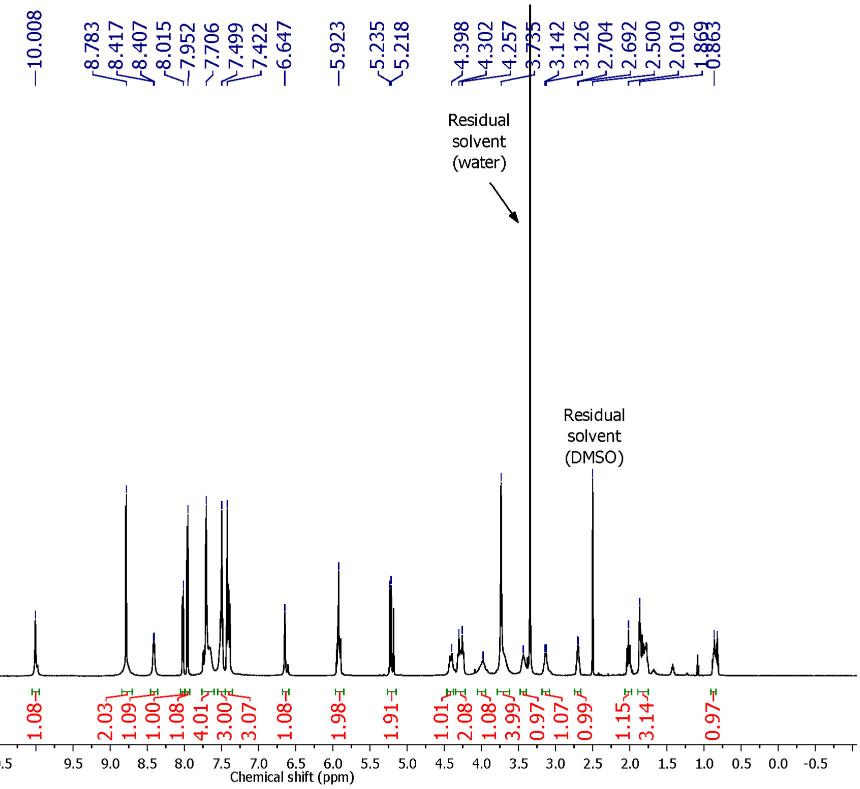

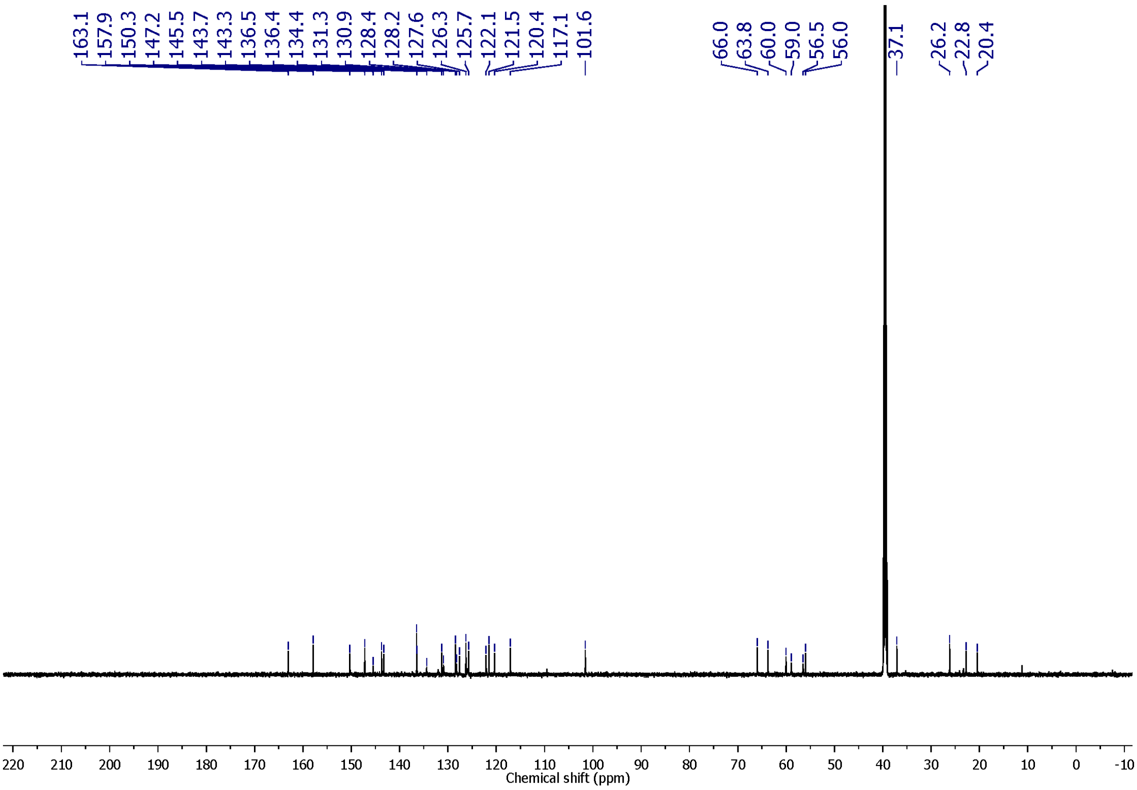

Figure S3. ^{1}H NMR (600 MHz) and ^{13}C NMR (151 MHz) spectra of compound **O** in DMSO-d_6.

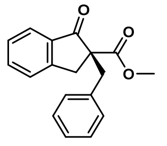

2a

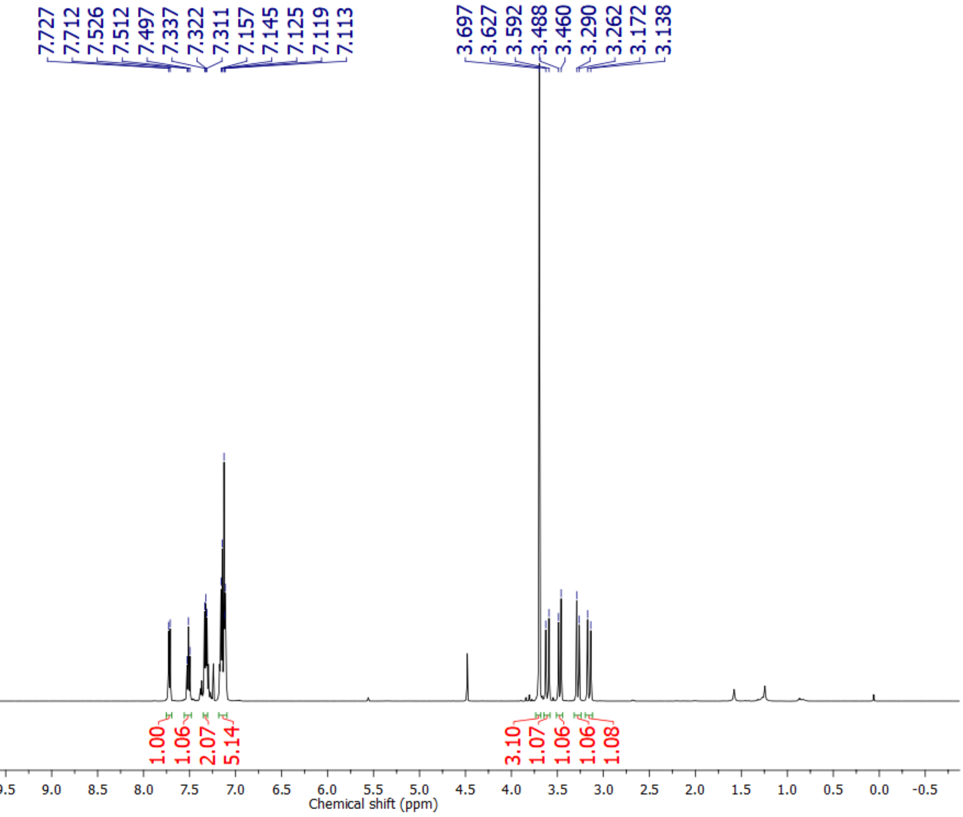
7.727
7.712
7.526
7.512
7.497
7.337
7.322
7.311
7.157
7.145
7.125
7.119
7.113
3.697
3.627
3.592
3.488
3.460
3.290
3.262
3.172
3.138
1.00
1.06
2.07
5.14
3.10
1.07
1.06
1.06
1.08
11.5 11.0 10.5 10.0 9.5 9.0 8.5 8.0 7.5 7.0 6.5 6.0 5.5 5.0 4.5 4.0 3.5 3.0 2.5 2.0 1.5 1.0 0.5 0.0 -0.5
Chemical shift (ppm)

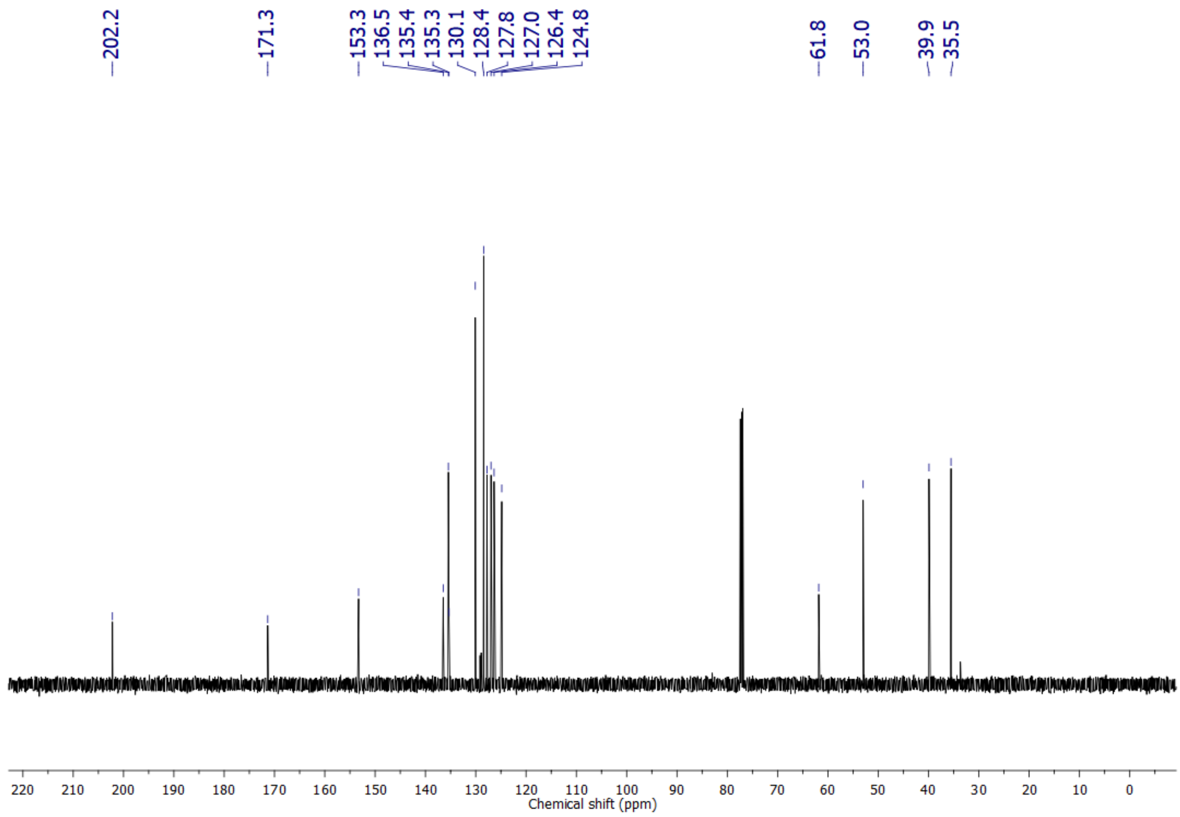

Figure S4. ^{1}H NMR (500 MHz) and ^{13}C NMR (126 MHz) spectra of compound **2a** in $CDCl_3$.

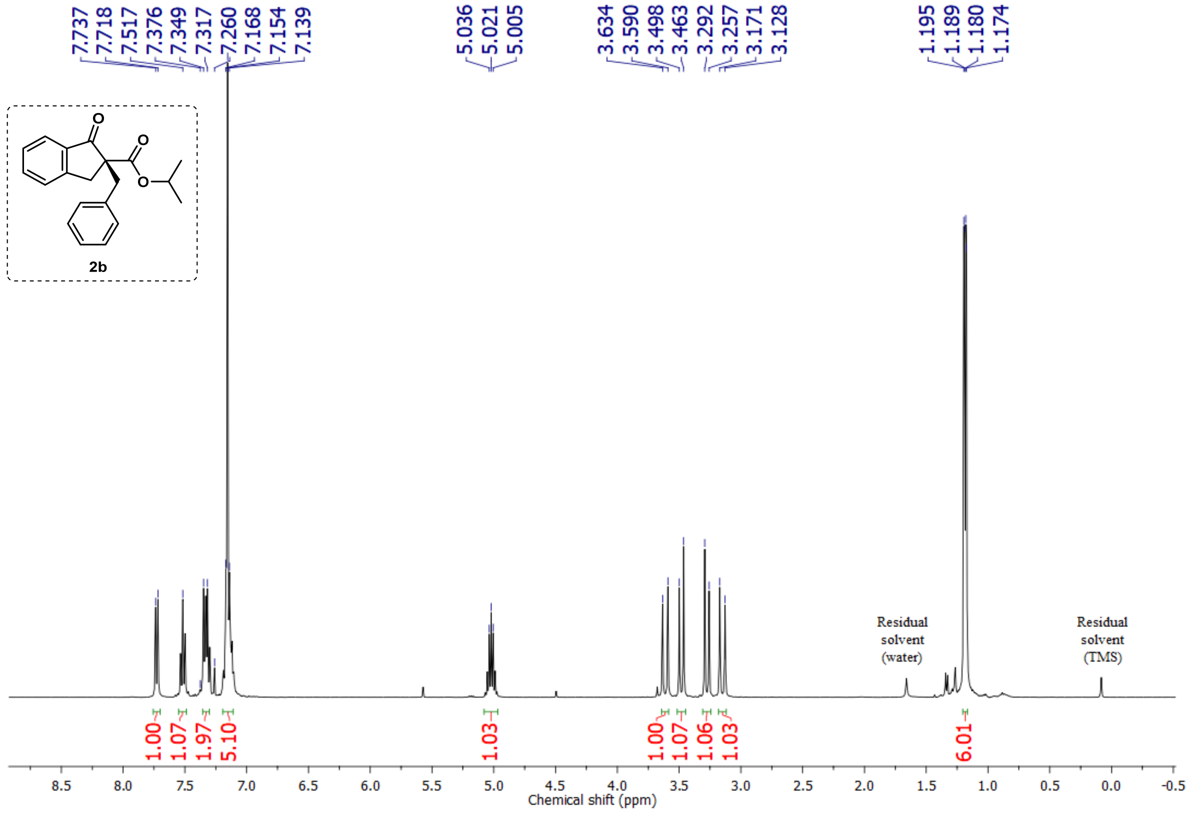

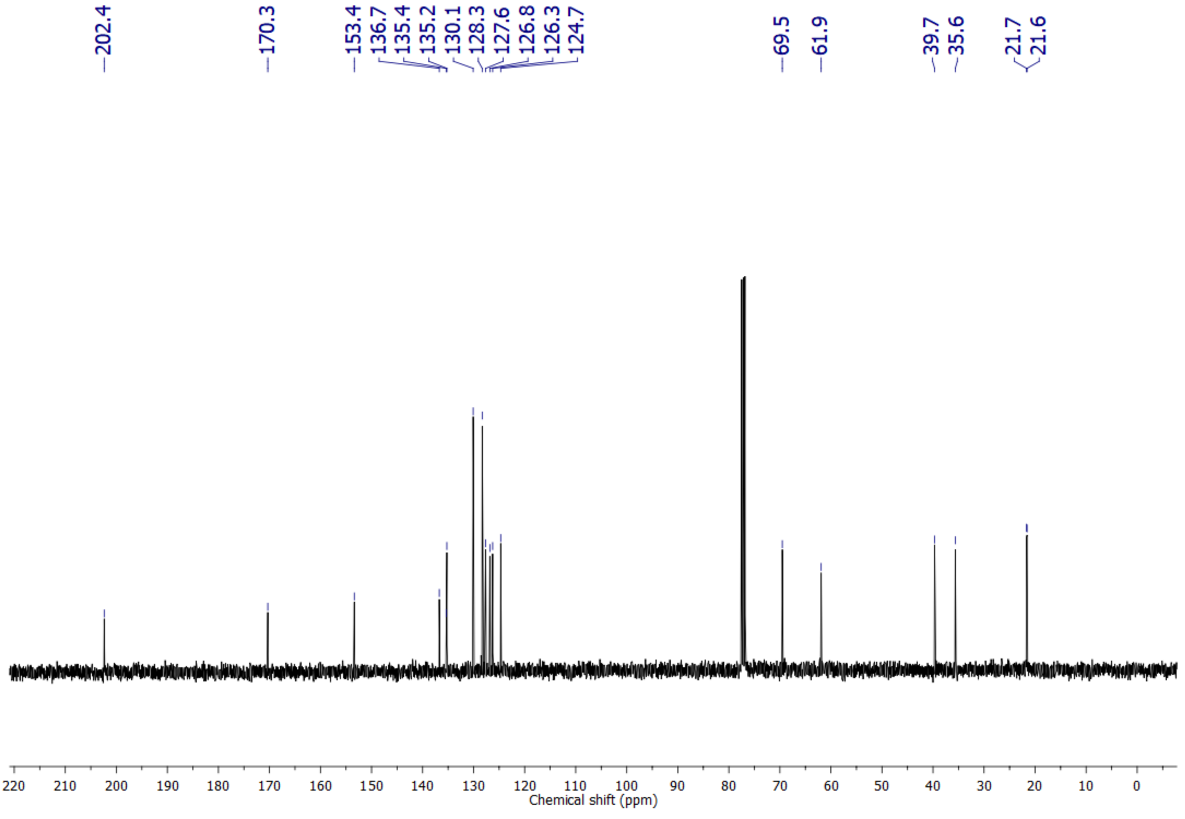

Figure S5. ^{1}H NMR (400 MHz) and ^{13}C NMR (101 MHz) spectra of compound **2b** in $CDCl_3$.

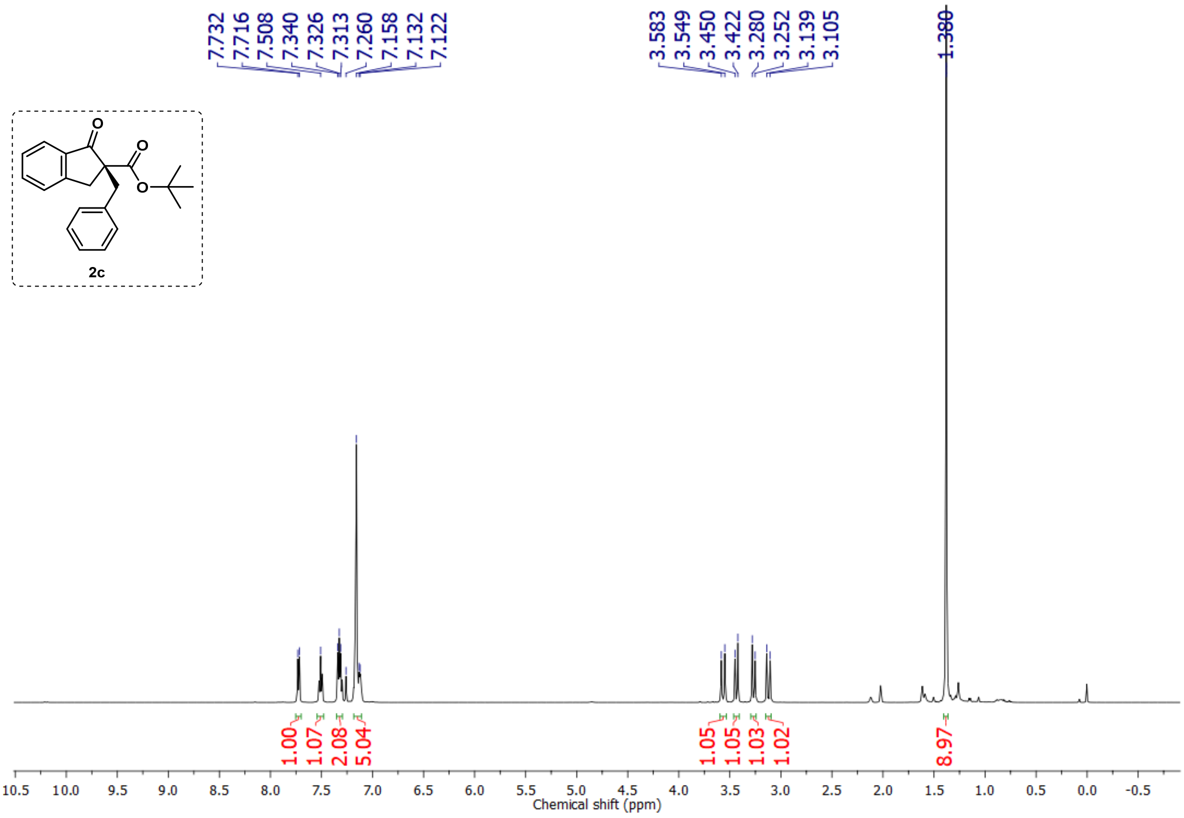

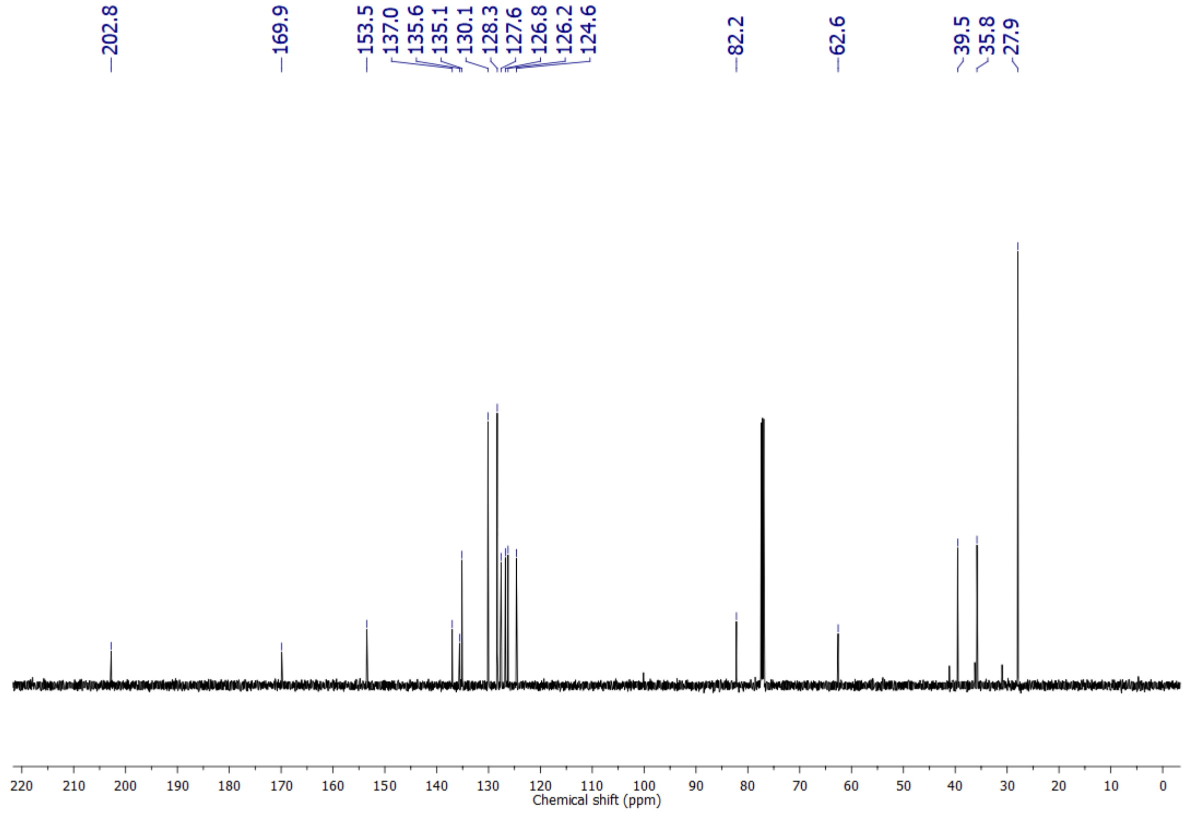

Figure S6. ^{1}H NMR (500 MHz) and ^{13}C NMR (126 MHz) spectra of compound **2c** in $CDCl_3$.

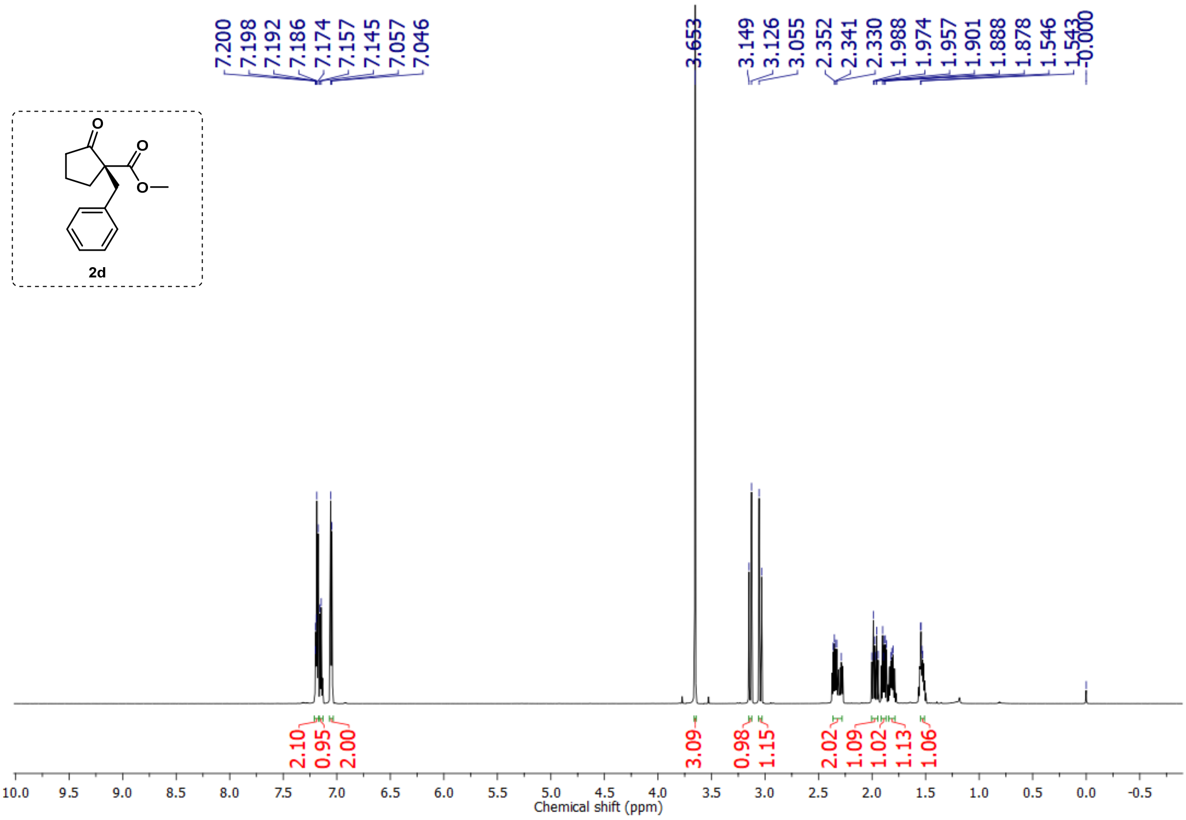

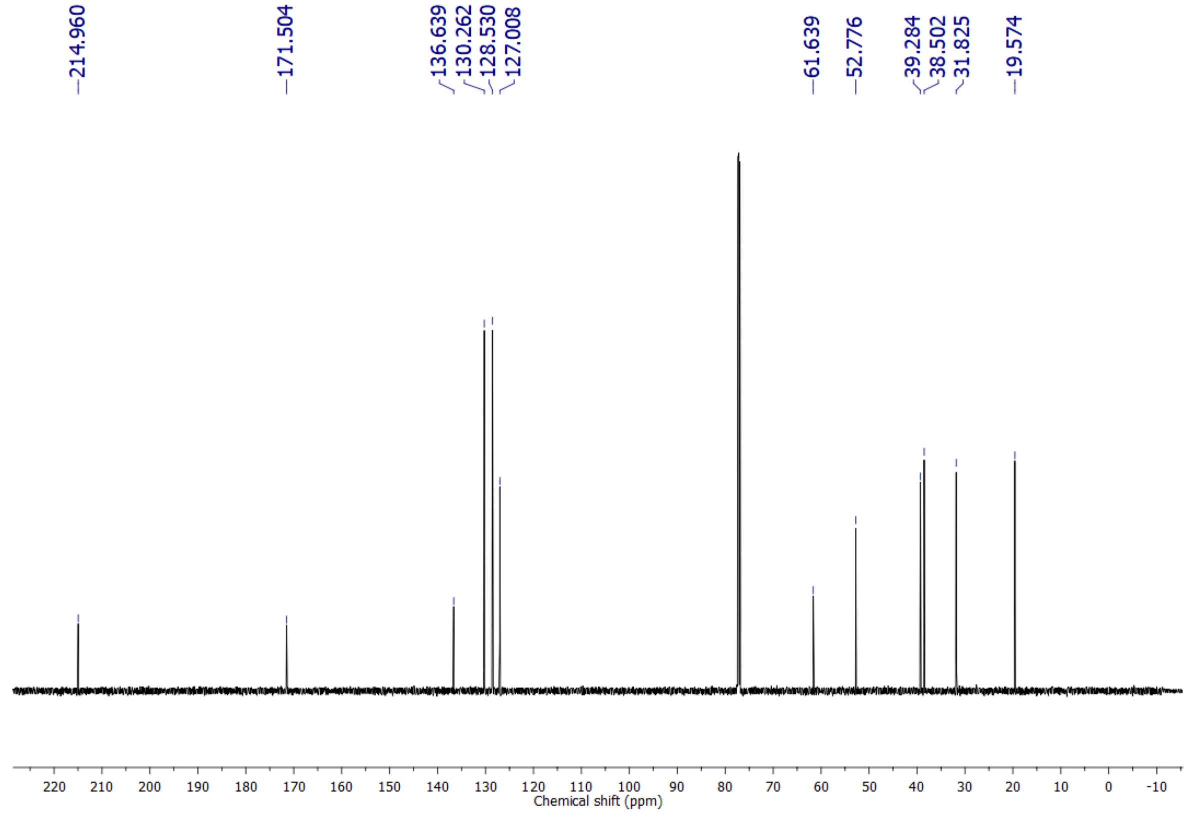

Figure S7. ^{1}H NMR (600 MHz) and ^{13}C NMR (151 MHz) spectra of compound **2d** in $CDCl_3$.

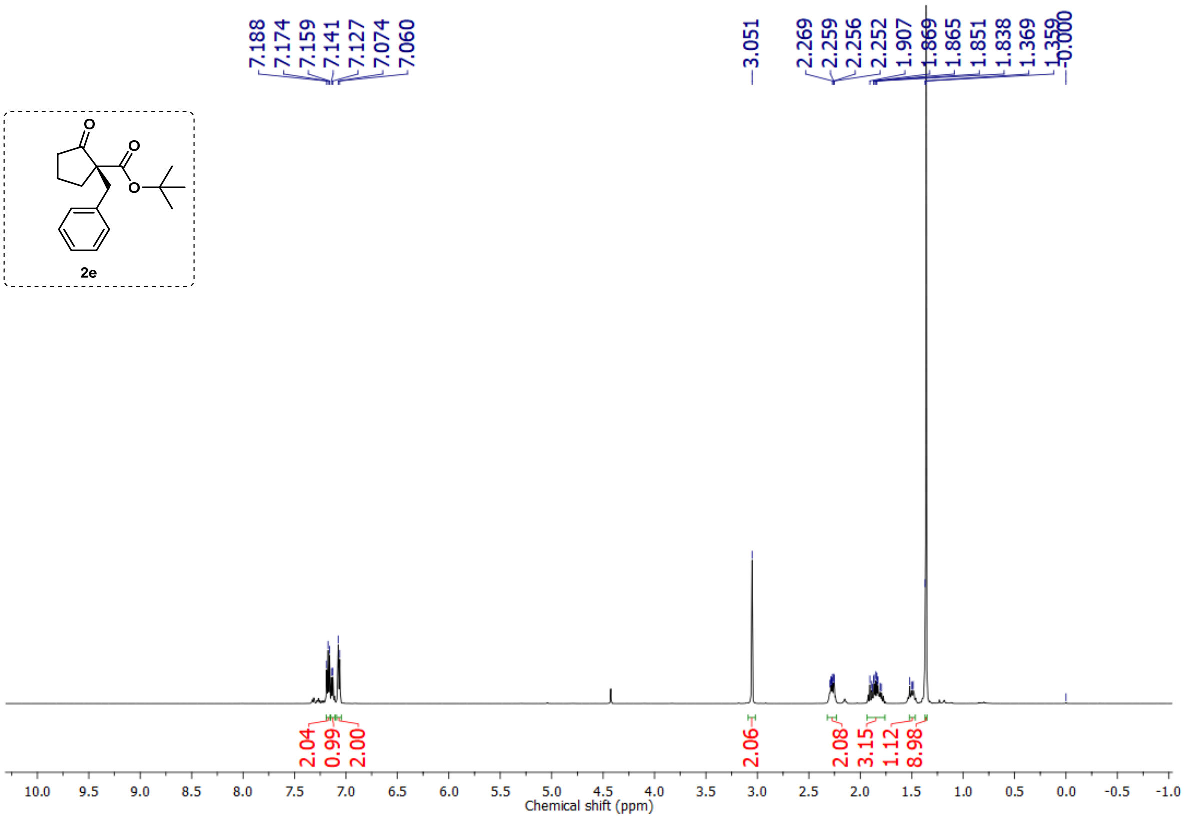

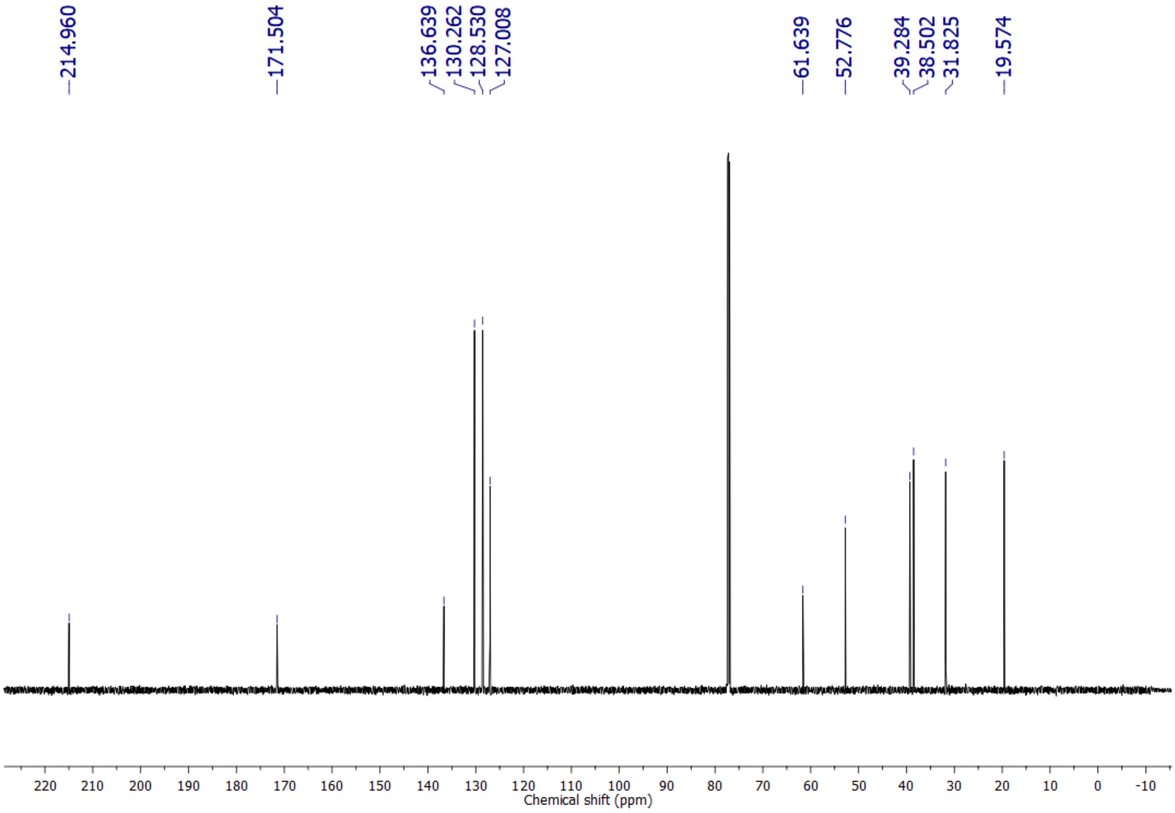

Figure S8. ^{1}H NMR (500 MHz) and ^{13}C NMR (126 MHz) spectra of compound **2e** in $CDCl_3$.

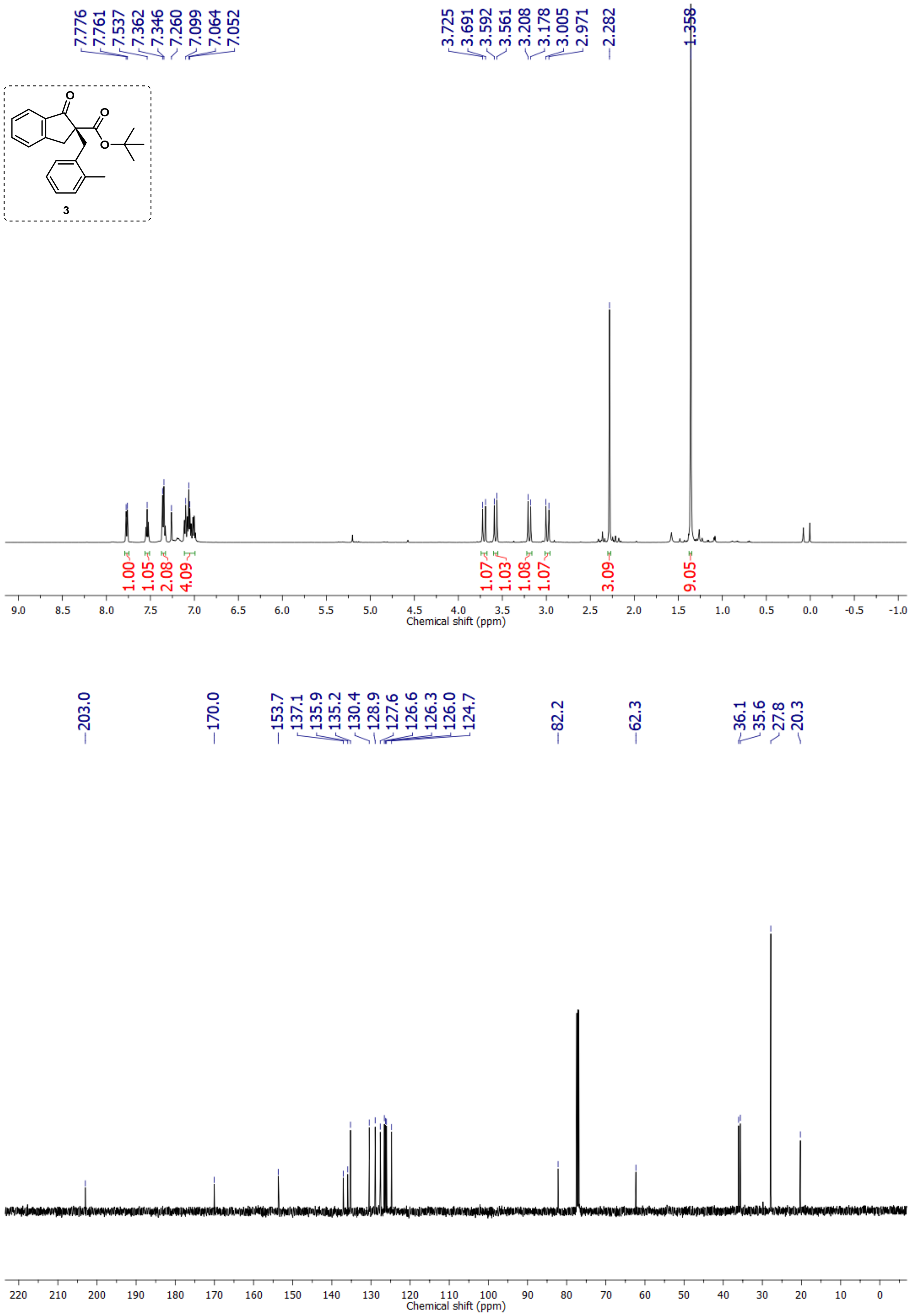

Figure S9. ^{1}H NMR (500 MHz) and ^{13}C NMR (126 MHz) spectra of compound **3** in $CDCl_3$.

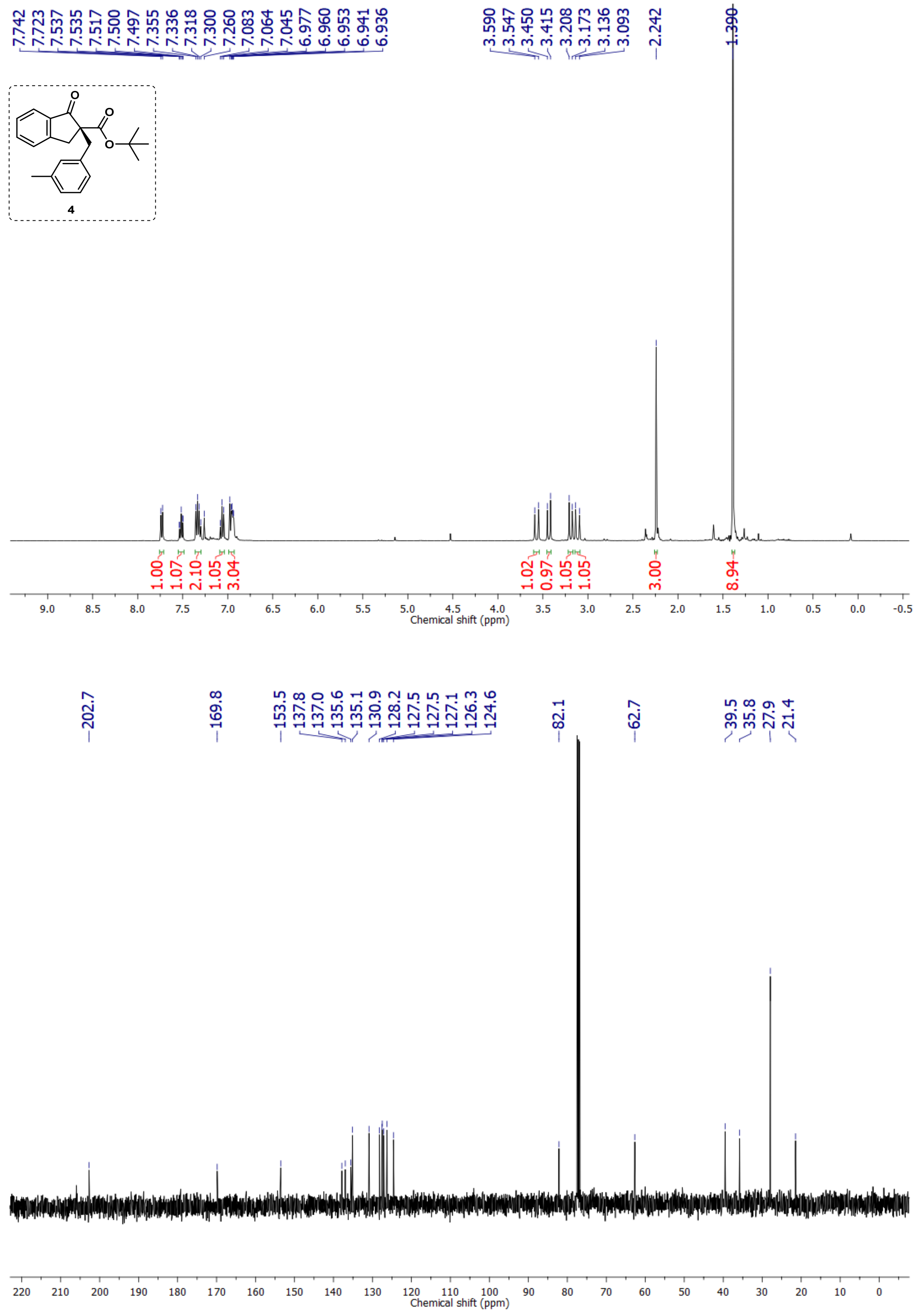

Figure S10. ^{1}H NMR (400 MHz) and ^{13}C NMR (101 MHz) spectra of compound **4** in $CDCl_3$.

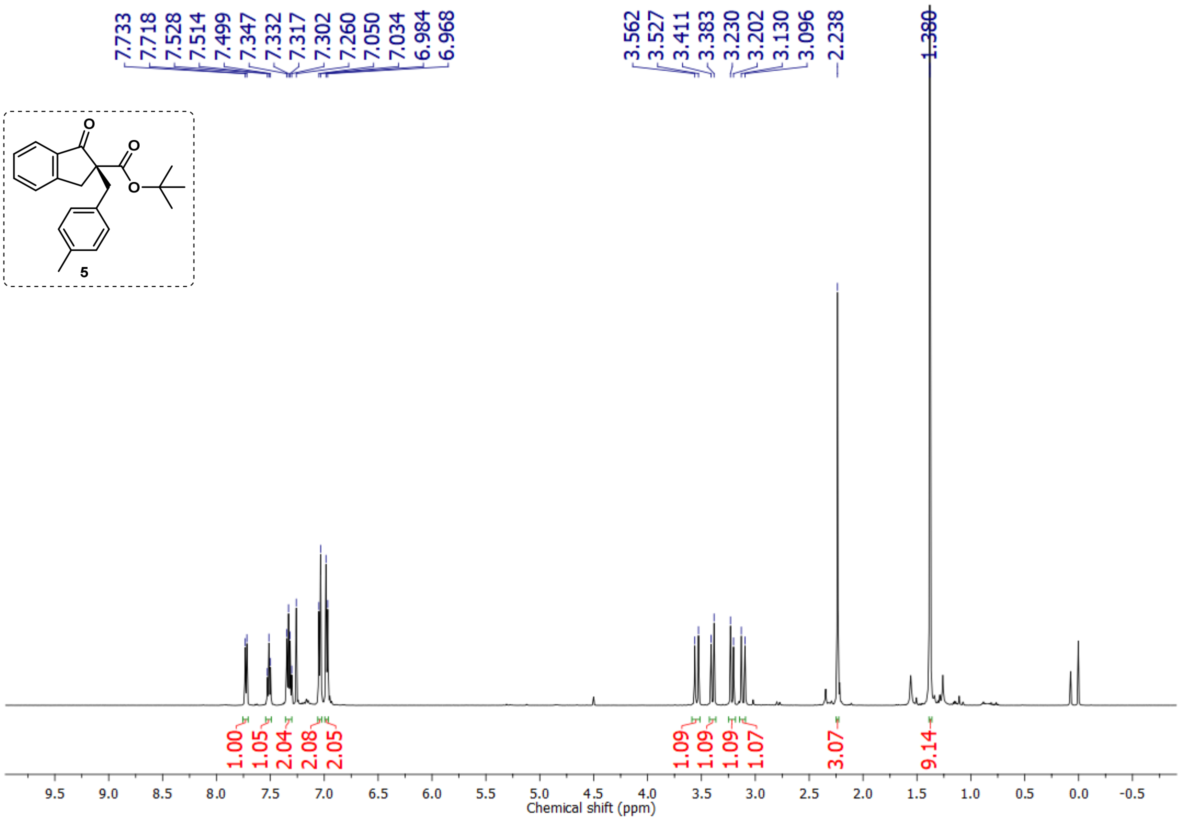

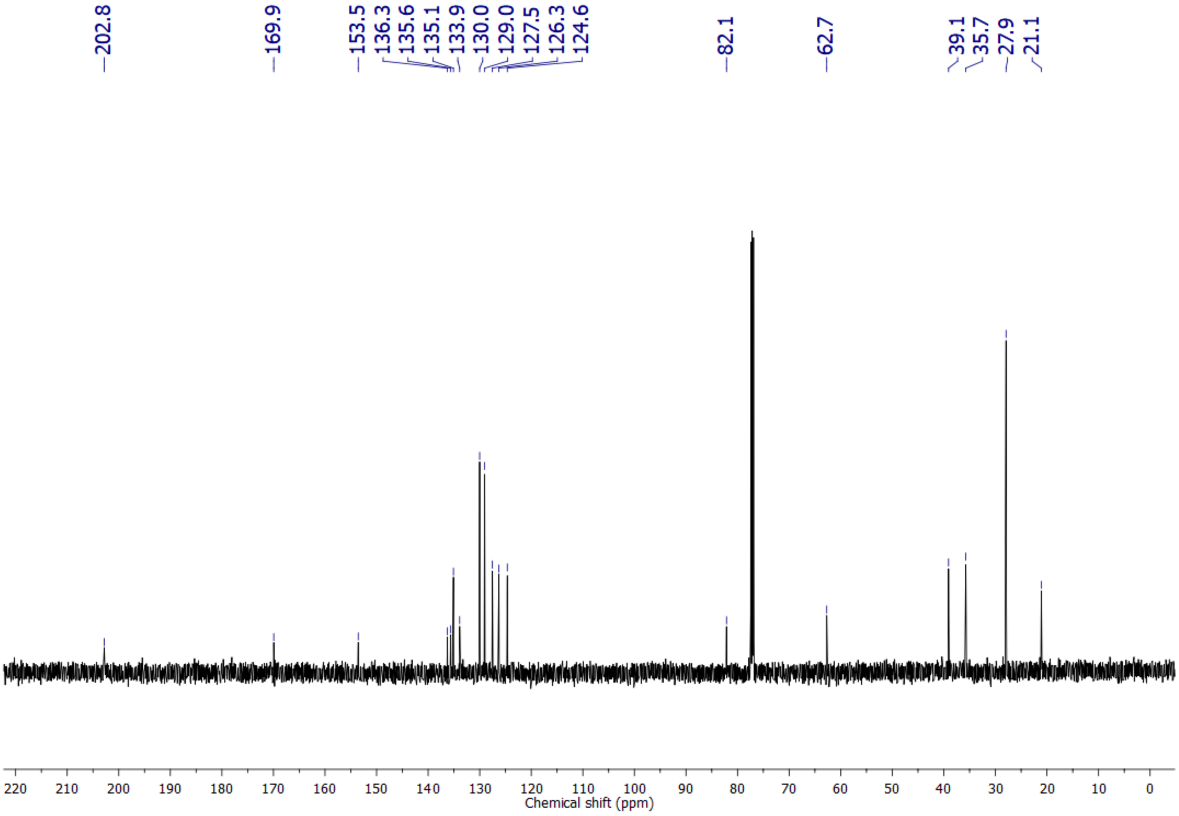

Figure S11. ^{1}H NMR (500 MHz) and ^{13}C NMR (126 MHz) spectra of compound **5** in $CDCl_3$.

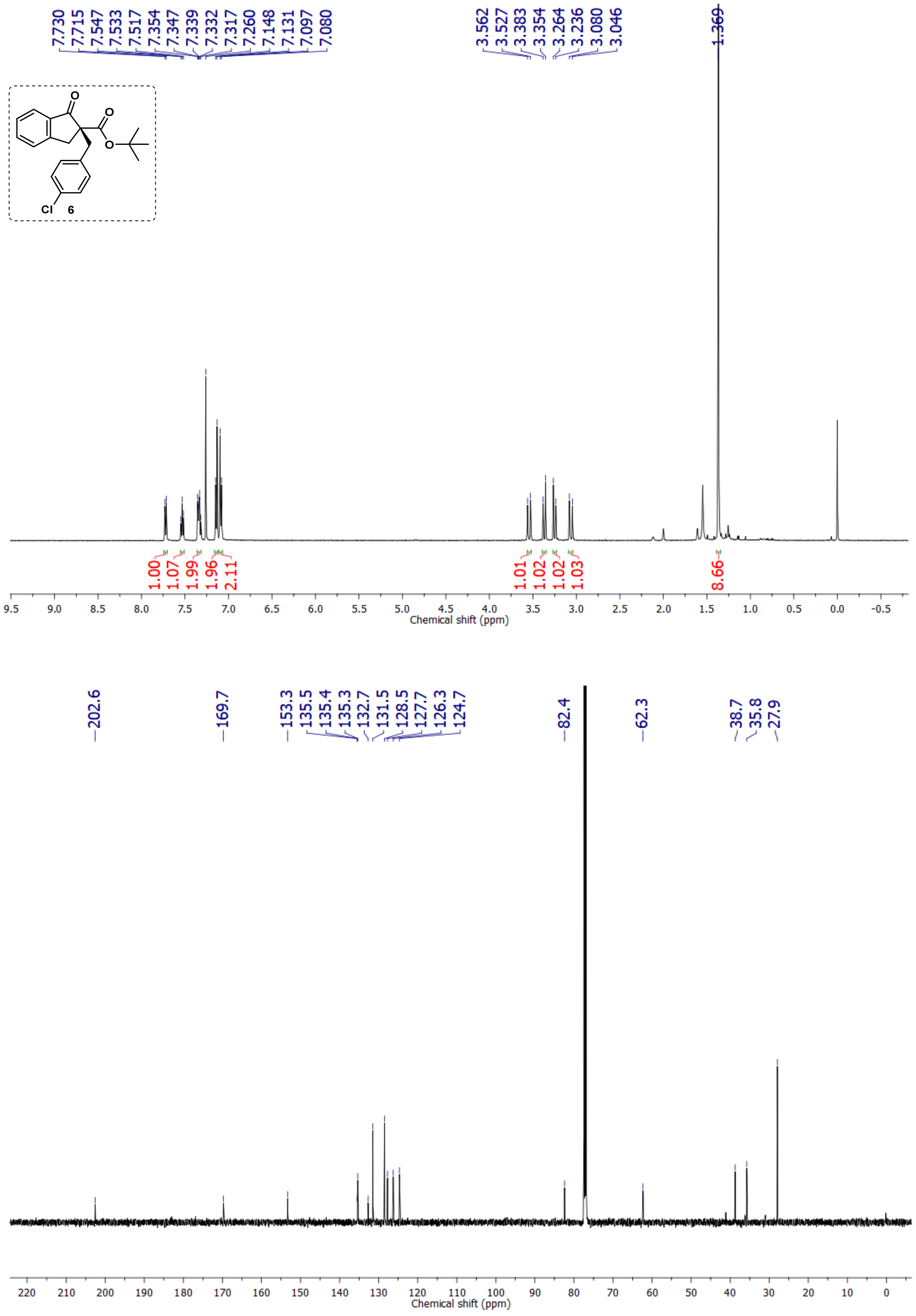

Figure S12. ^{1}H NMR (500 MHz) and ^{13}C NMR (126 MHz) spectra of compound **6** in $CDCl_3$.

2. DFT studies and corresponding Cartesian coordinates

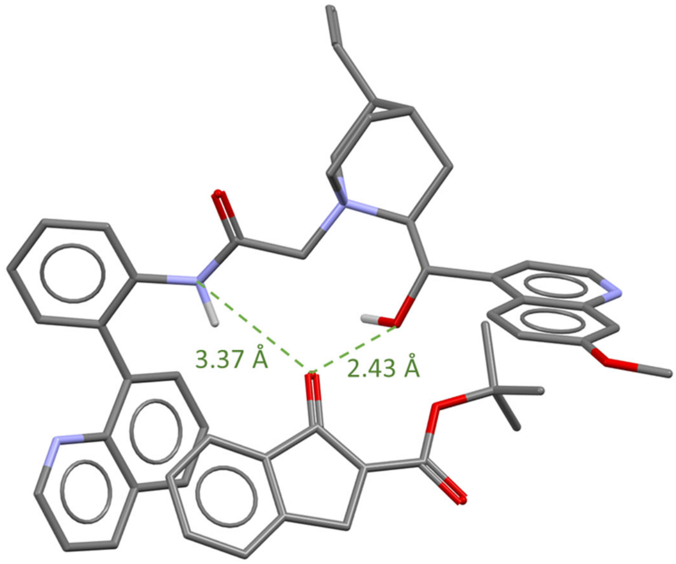

Figure S13. Model of a possible intermediate state for the reaction obtained at DFT/M06-2X/6-31G(d) level of theory using program Spartan'18 Parallel Suite.[1-6]

Table S1. Model of a possible intermediate state for the reaction.

E = -5218.140044 au							
H	-3.561144	-3.756491	-2.091655	C	2.376937	-0.671837	-0.621128
C	-4.453537	-3.229913	-1.694378	H	3.757538	-1.365639	1.652243
C	-6.636890	-1.821094	-0.696334	C	4.292216	-0.647644	1.002031
C	-4.243047	-2.165972	-0.806983	C	3.759346	-0.220850	-0.188901
C	-5.741499	-3.583276	-2.068629	N	6.305756	0.628479	0.658867
C	-6.834757	-2.885360	-1.567892	C	4.518374	0.676586	-1.005882
C	-5.349372	-1.449775	-0.309564	C	5.579894	-0.195953	1.398851
H	-5.892355	-4.413333	-2.765528	C	5.794816	1.080463	-0.555181
H	-7.849372	-3.165028	-1.866482	C	4.049667	1.183266	-2.247850
H	-7.495695	-1.258872	-0.314880	H	6.027990	-0.522550	2.344908
C	-4.342560	-0.407912	1.687954	H	7.581256	2.255083	-0.950865
C	-5.184563	-0.306015	0.603942	C	4.812750	2.032275	-2.998991
C	-4.791382	1.875852	2.338169	H	3.045449	0.884077	-2.587251
C	-5.885947	0.916537	0.373886	H	4.440646	2.422790	-3.952994
C	-4.151235	0.685296	2.560349	C	6.101706	2.421745	-2.532557
C	-5.669103	2.015342	1.234034	C	6.595066	1.962172	-1.331700
N	-6.762240	1.026652	-0.703422	O	6.758681	3.272215	-3.402572
H	-3.466212	0.572974	3.406969	C	8.006619	3.772248	-2.992352
H	-6.120851	4.119619	1.579598	H	7.939738	4.352160	-2.064464
H	-4.623716	2.737603	2.994877	H	8.282506	4.425471	-3.824332
C	-7.359698	2.181298	-0.947627	H	8.753415	2.978108	-2.875445
H	-8.015025	2.210595	-1.825899	C	-2.081845	2.666613	-0.735585
C	-7.149778	3.333898	-0.138003	C	-3.858804	4.306598	-2.085063
H	-7.631141	4.276727	-0.416070	C	-1.947948	4.066067	-0.670762
C	-6.321949	3.248999	0.944610	C	-3.102541	2.088778	-1.469717
H	-3.798976	-1.340323	1.876136	C	-3.993170	2.924012	-2.147411
N	-2.922693	-1.836803	-0.338651	C	-2.832213	4.892940	-1.343062
H	-2.830372	-0.909248	0.024995	H	-3.206878	0.999923	-1.508691
C	-1.738063	-2.389483	-0.862725	H	-4.807085	2.484856	-2.731754
O	-1.768947	-3.324696	-1.673991	H	-2.727647	5.980290	-1.293802
C	-0.443259	-1.817959	-0.318465	H	-4.567129	4.944407	-2.622308
H	-0.515906	-1.555988	0.771784	C	-0.987669	2.032184	0.084328
H	-0.277408	-0.763602	-0.833831	C	-0.774408	4.425542	0.187362
H	3.704665	-3.210530	0.802201	H	-0.034411	5.025700	-0.375403
C	3.231276	-3.000901	-0.177653	H	-1.087641	5.043141	1.051244
C	1.754051	-4.971375	0.094696	C	-0.219622	3.095613	0.616434
N	0.770347	-2.705957	-0.510715	O	-0.899285	0.795078	0.212844
C	0.567068	-4.011504	0.274055	C	0.948864	3.073090	1.436481
C	2.047556	-2.043081	0.028755	O	1.399886	1.810062	1.758511

C	2.776870	-4.301969	-0.835136	C	2.629293	1.576282	2.465087
H	0.435279	-3.758913	1.355781	C	2.620675	2.139697	3.889658
H	1.860329	-1.870983	1.210770	H	2.727908	3.232042	3.900452
H	3.649077	-4.977905	-0.985333	H	3.449948	1.720027	4.474435
H	2.232357	-5.139218	1.092060	H	1.684640	1.892533	4.412686
H	-0.379173	-4.497201	-0.047655	C	3.858728	2.081562	1.701258
C	0.951810	-3.037855	-1.986736	H	4.779643	1.786049	2.220901
H	1.105855	-2.090918	-2.549961	H	3.866704	3.174956	1.607191
H	-0.034199	-3.455592	-2.350360	H	3.903063	1.659325	0.686965
C	2.120120	-3.998960	-2.182880	C	2.689510	0.038948	2.536486
H	2.859785	-3.574582	-2.887250	H	1.834300	-0.377513	3.085544
H	1.760282	-4.948720	-2.636778	H	3.604173	-0.296985	3.041765
H	4.023405	-2.528514	-0.796383	H	2.686396	-0.425825	1.532087
C	1.292069	-6.279424	-0.465335	O	1.564498	4.045308	1.883365
H	1.094941	-6.277016	-1.550236	Br	1.512506	-1.630469	2.760558
C	1.128682	-7.366275	0.278137	H	2.320840	-0.750974	-1.742061
H	1.318335	-7.391442	1.348120	O	1.402924	0.302681	-0.371899
H	0.786166	-8.309264	-0.136429	H	0.613128	-0.107755	-0.014227

3. Copies of HPLC chromatograms

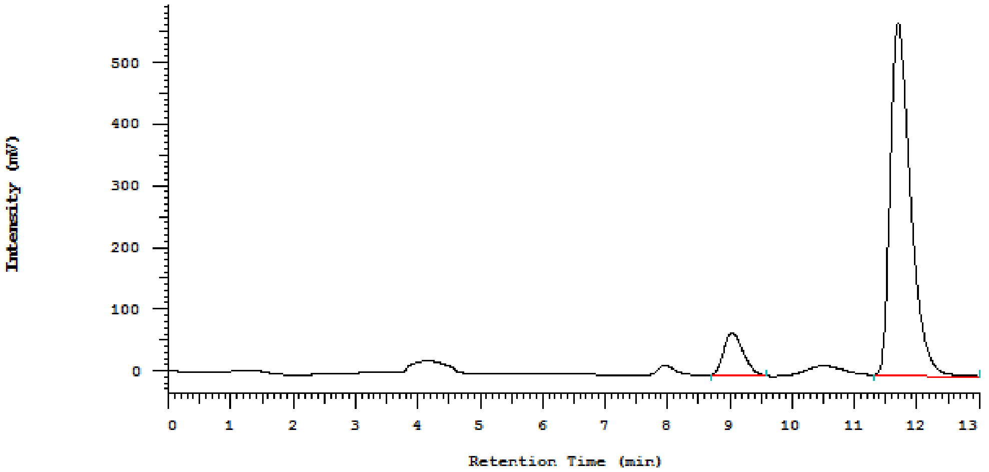

No.	RT	Area	Height	Area %
1	9,040	1509962	69494	10,558
2	11,700	12791630	570187	89,442
		14301592	639681	100,000

Figure S14. Chromatogram of product **2a.**

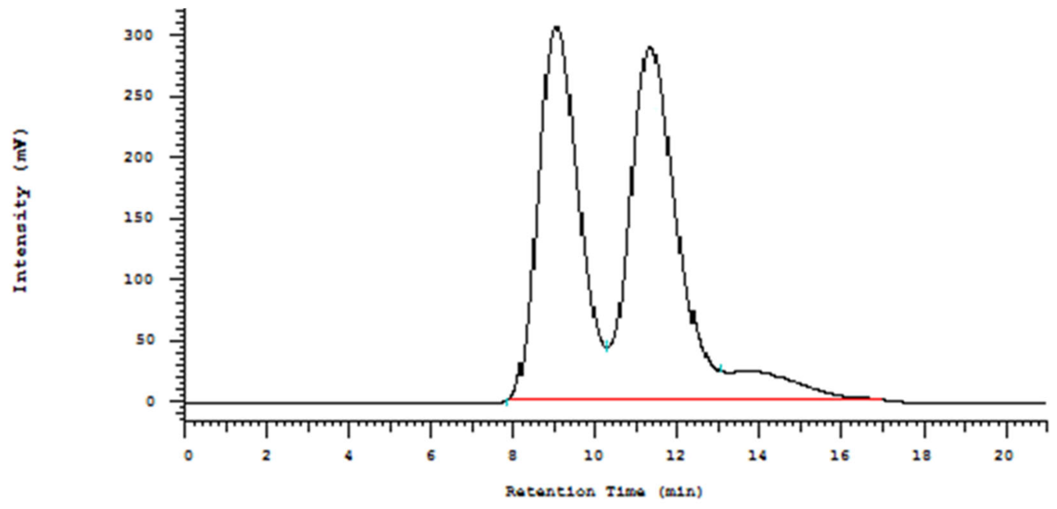

No.	RT	Area	Conc 1
1	9,060	15145753	49,534
2	11,727	15430429	50,466
		30576182	100,000

Figure S15. Chromatogram of product **2a** racemate.

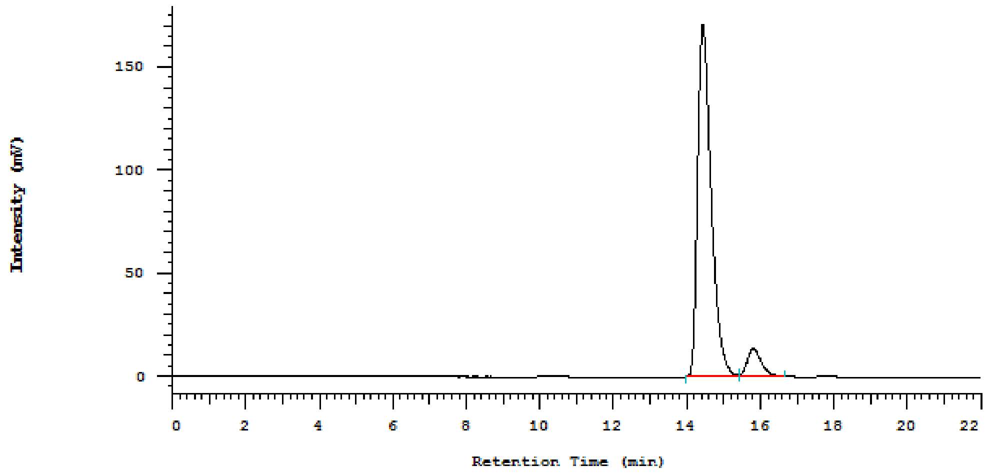

No.	RT	Area	Height	Area %
1	14,433	4372392	170820	92,306
2	15,800	364465	13593	7,694
		4736857	184413	100,000

Figure S16. Chromatogram of product **2b** .

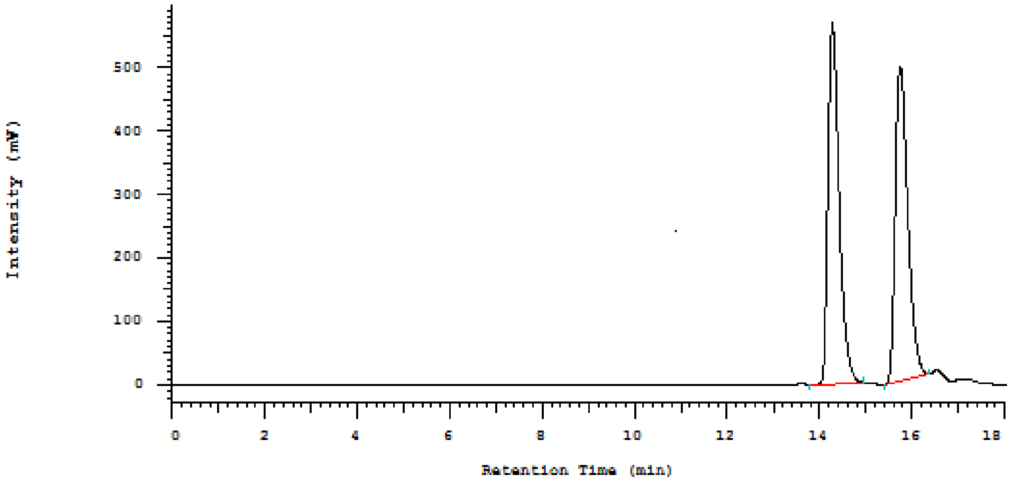

No.	RT	Area	Height	Area %
1	14,227	9433286	566661	50,404
2	15,893	9282210	493649	49,596
		18715496	1060310	100,000

Figure S17. Chromatogram of product **2b** racemate.

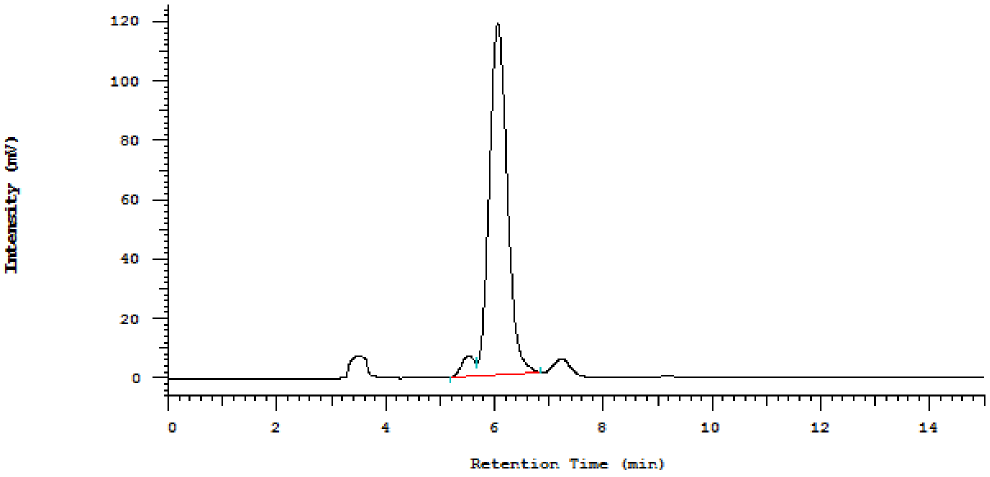

No.	RT	Area	Height	Area %
1	5,520	117769	7041	4,151
2	6,060	2719213	118456	95,849
		2836982	125497	100,000

Figure S18. Chromatogram of product **2c.**

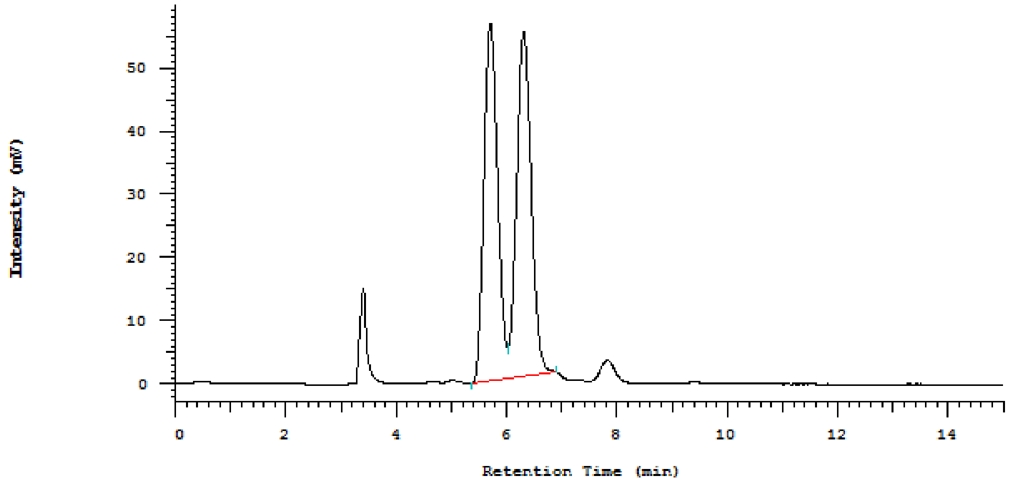

No.	RT	Area	Height	Area %
1	5,707	970179	56507	49,177
2	6,307	1002662	54473	50,823
		1972841	110980	100,000

Figure S19. Chromatogram of product **2c** racemate .

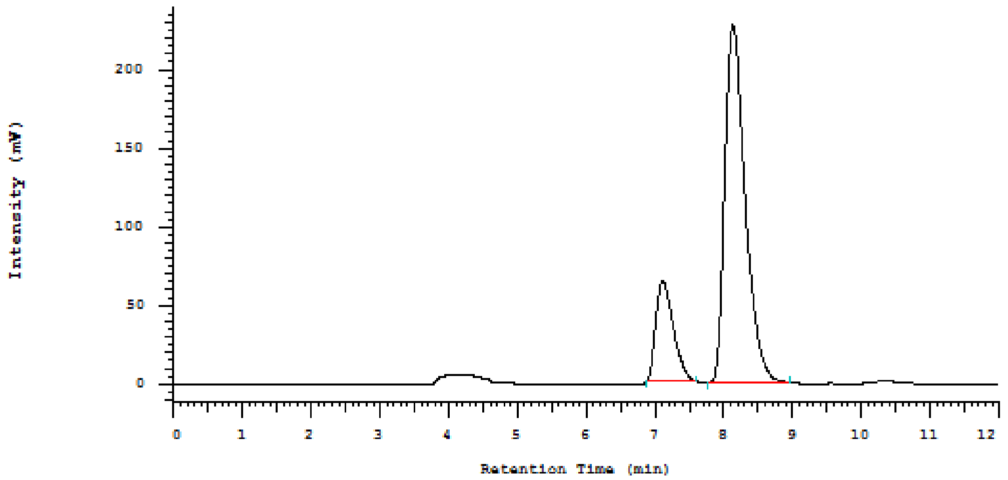

No.	RT	Area	Height	Area %
1	7,113	1132049	63629	19,560
2	8,133	4655598	227080	80,440
		5787647	290709	100,000

Figure S20. Chromatogram of product **2d.**

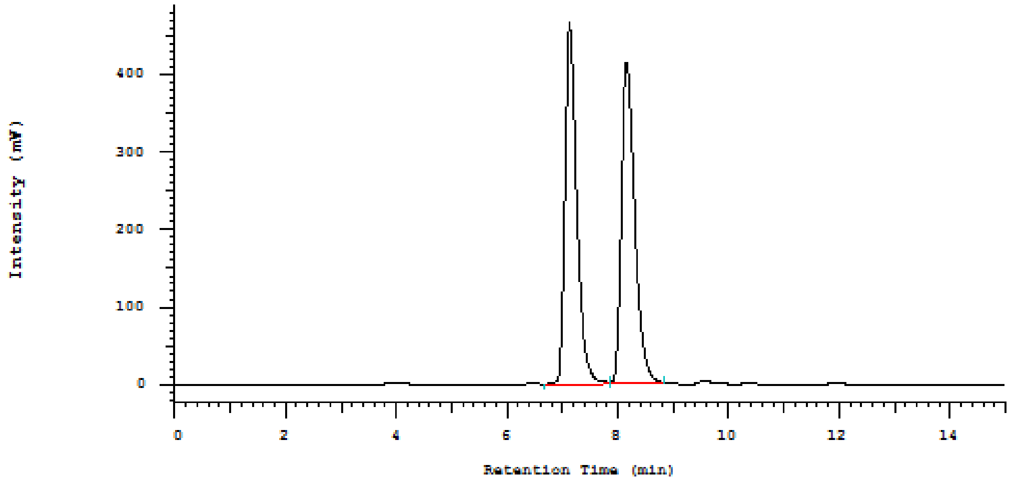

No.	RT	Area	Height	Area %
1	7,127	6910918	464327	50,232
2	8,160	6847153	412995	49,768
		13758071	877322	100,000

Figure S21. Chromatogram of product **2d** racemate .

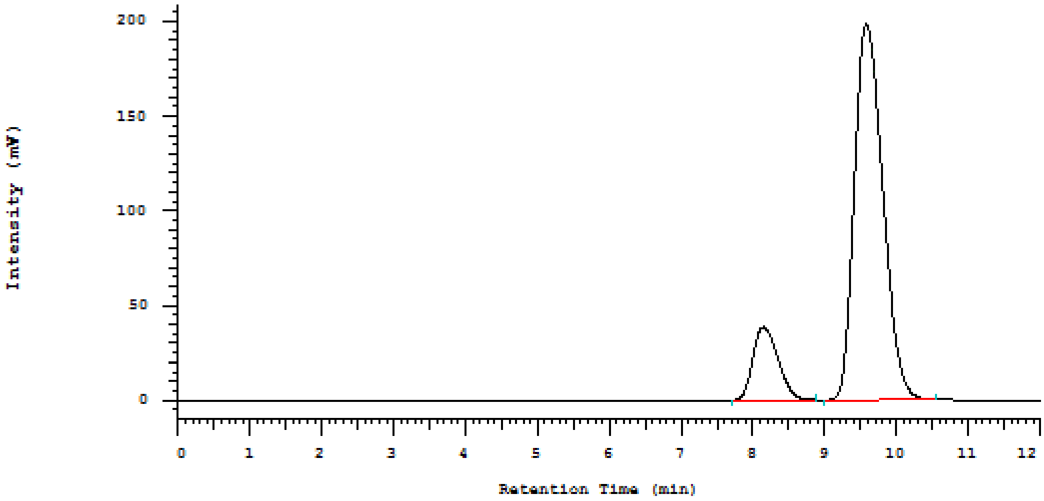

No.	RT	Area	Conc 1
1	8,240	802352	12,779
2	9,580	5476464	87,221
		6278816	100,000

Figure S22. Chromatogram of product **2e.**

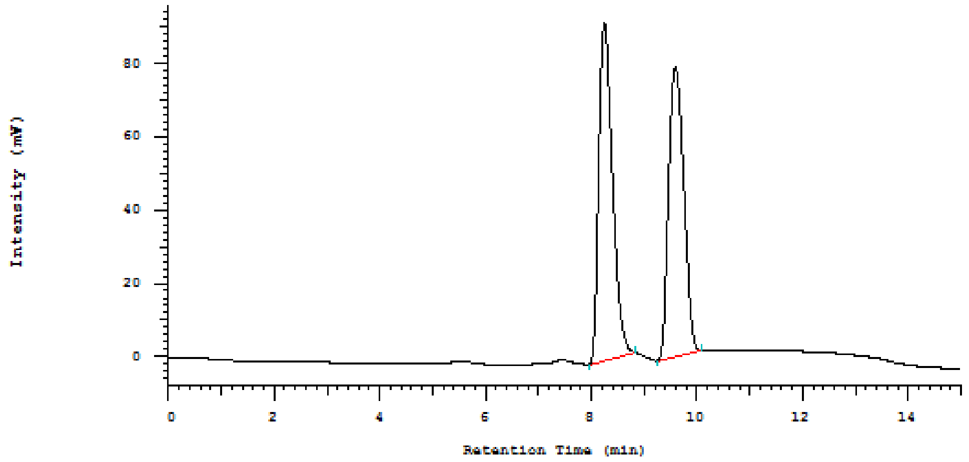

No.	RT	Area	Height	Area %
1	8,247	1647596	91854	51,065
2	9,593	1578900	78896	48,935
		3226496	170750	100,000

Figure S23. Chromatogram of product **2e** racemate.

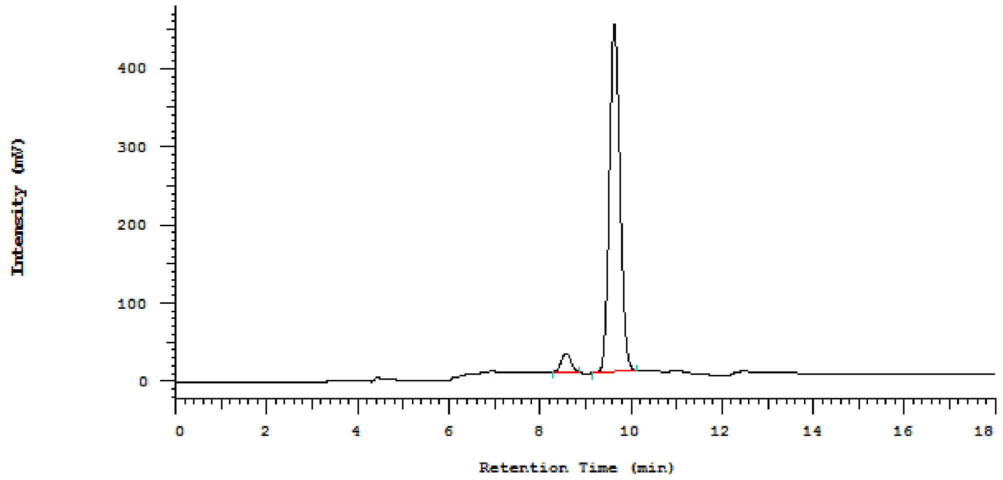

No.	RT	Area	Height	Area %
1	8,573	359816	24170	4,739
2	9,640	7232336	444382	95,261
		7592152	468552	100,000

Figure S24. Chromatogram of product **3.**

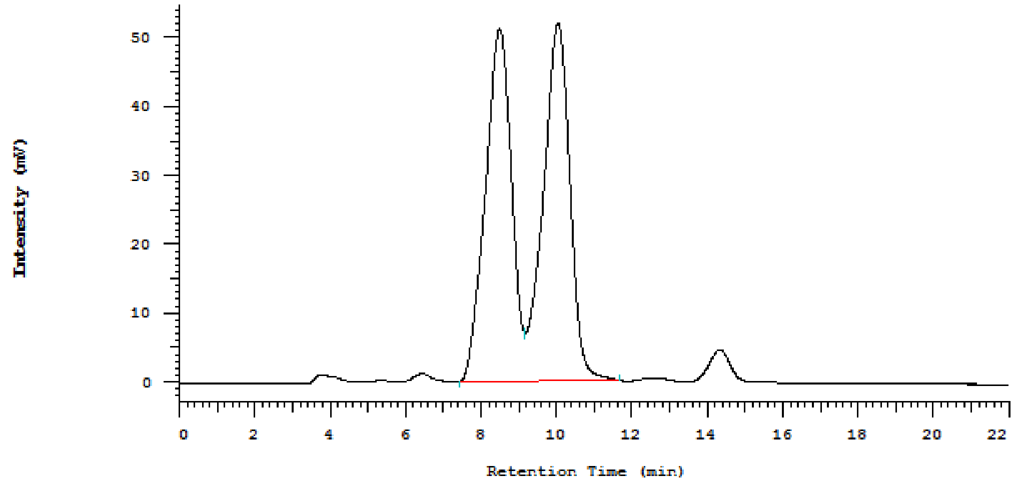

No.	RT	Area	Height	Area %
1	8,493	2467540	51176	49,204
2	10,040	2547370	51878	50,796
		5014910	103054	100,000

Figure S25. Chromatogram of product **3** racemate.

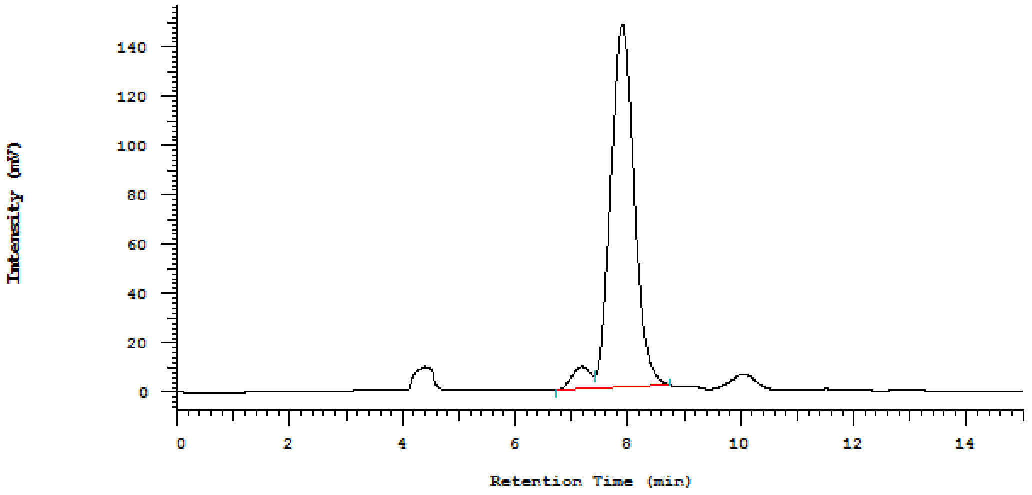

No.	RT	Area	Height	Area %
1	7,180	205703	9046	4,608
2	7,900	4258570	147169	95,392
		4464273	156215	100,000

Figure S26. Chromatogram of product **4.**

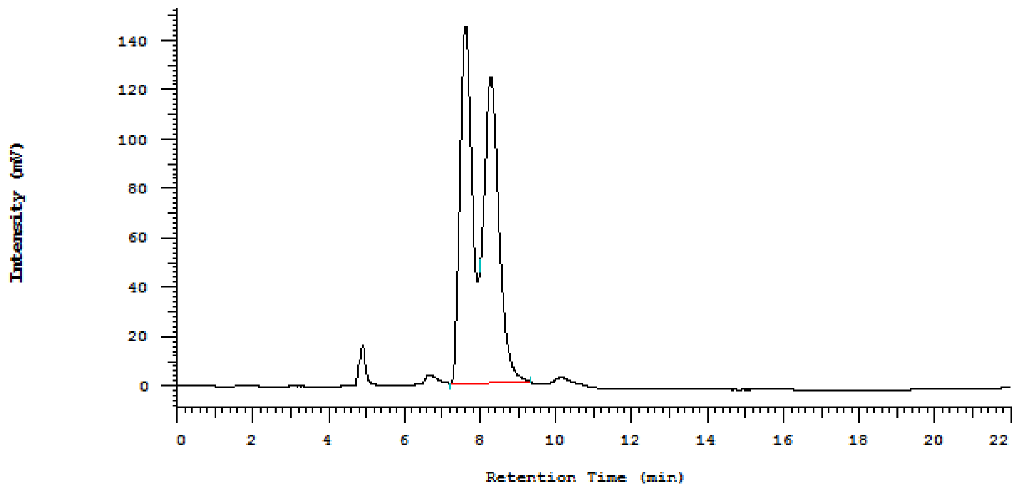

No.	RT	Area	Height	Area %
1	7,613	3367044	144281	49,684
2	8,287	3409916	123875	50,316
		6776960	268156	100,000

Figure S27. Chromatogram of product **4** racemate.

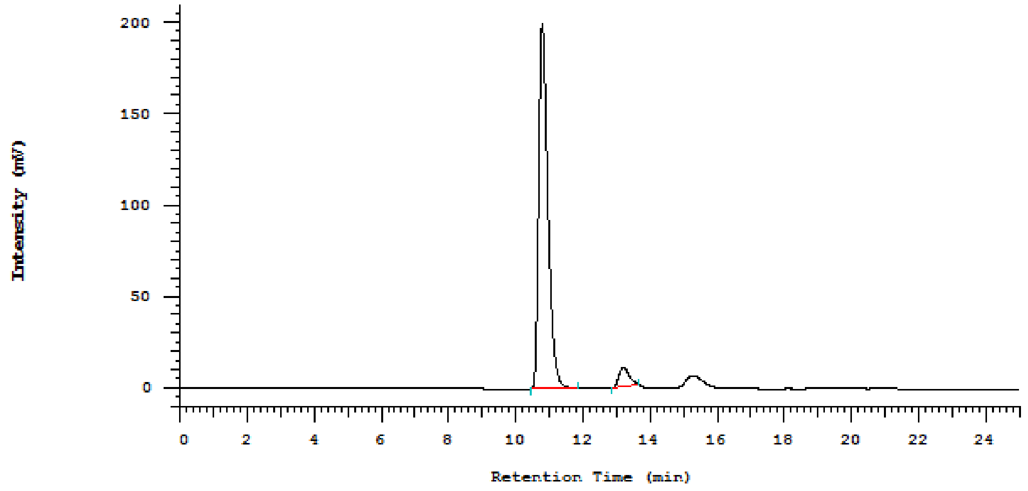

No.	RT	Area	Height	Area %
1	10,793	3800488	199722	94,475
2	13,207	222252	10624	5,525
		4022740	210346	100,000

Figure S28. Chromatogram of product **5.**

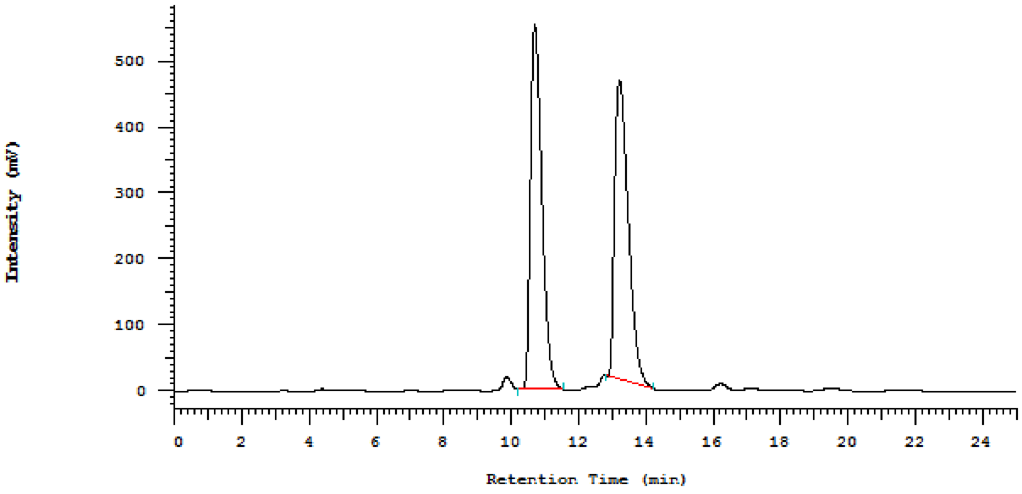

No.	RT	Area	Height	Area %
1	10,707	13056704	552436	50,673
2	13,220	12709989	452387	49,327
		25766693	1004823	100,000

Figure S29. Chromatogram of product **5** racemate.

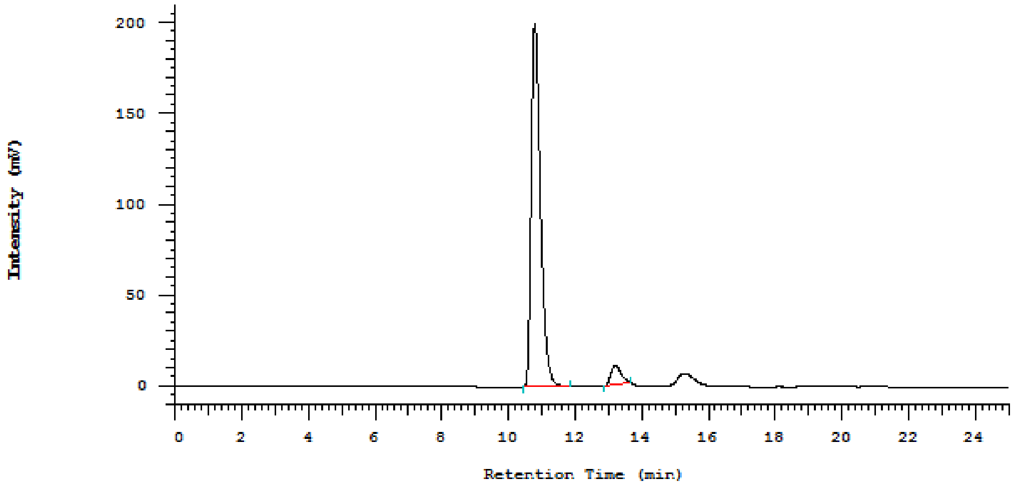

No.	RT	Area	Height	Area %
1	10,793	3800488	199722	94,475
2	13,207	222252	10624	5,525
		4022740	210346	100,000

Figure S30. Chromatogram of product **6**.

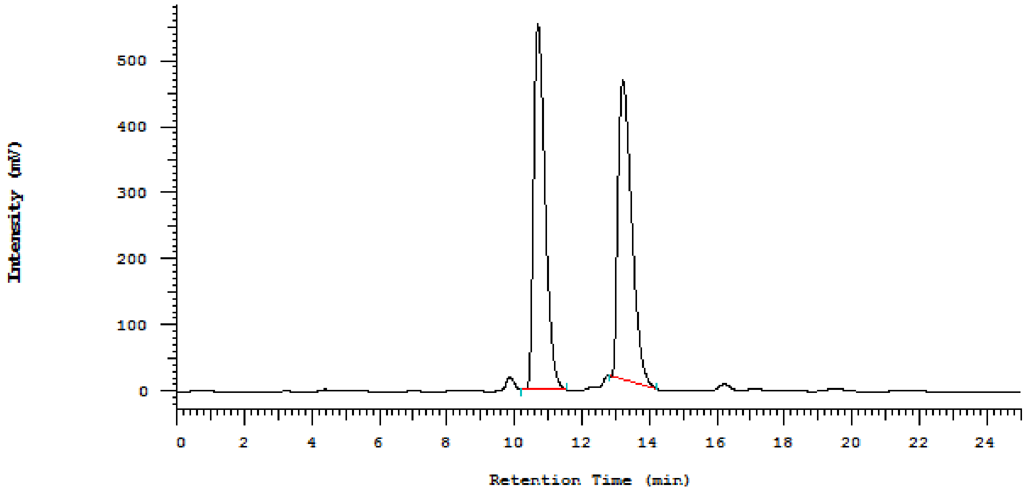

No.	RT	Area	Height	Area %
1	10,707	13056704	552436	50,673
2	13,220	12709989	452387	49,327
		25766693	1004823	100,000

Figure S31. Chromatogram of product **6** racemate.
